# Fibroblastic reticular cells in lymph node potentiate white adipose tissue beiging through neuro-immune crosstalk in male mice

**DOI:** 10.1038/s41467-023-36737-0

**Published:** 2023-03-03

**Authors:** Lai Yee Cheong, Baile Wang, Qin Wang, Leigang Jin, Kelvin H. M. Kwok, Xiaoping Wu, Lingling Shu, Huige Lin, Sookja Kim Chung, Kenneth K. Y. Cheng, Ruby L. C. Hoo, Aimin Xu

**Affiliations:** 1 State Key Laboratory of Pharmaceutical Biotechnology, The University of Hong Kong, Hong Kong, China; 2 Department of Medicine, The University of Hong Kong, Hong Kong, China; 3 Department of Pharmacology & Pharmacy, The University of Hong Kong, Hong Kong, China; 4 Department of Health Technology and Informatics, The Hong Kong Polytechnic University, Hong Kong, China; 5 School of Biomedical Sciences, The University of Hong Kong, Hong Kong, China; 6 Faculty of Medicine, Macau University of Science and Technology, Macau, China

**Keywords:** Lymph node, Obesity, Molecular medicine

## Abstract

Lymph nodes (LNs) are always embedded in the metabolically-active white adipose tissue (WAT), whereas their functional relationship remains obscure. Here, we identify fibroblastic reticular cells (FRCs) in inguinal LNs (iLNs) as a major source of IL-33 in mediating cold-induced beiging and thermogenesis of subcutaneous WAT (scWAT). Depletion of iLNs in male mice results in defective cold-induced beiging of scWAT. Mechanistically, cold-enhanced sympathetic outflow to iLNs activates β1- and β2-adrenergic receptor (AR) signaling in FRCs to facilitate IL-33 release into iLN-surrounding scWAT, where IL-33 activates type 2 immune response to potentiate biogenesis of beige adipocytes. Cold-induced beiging of scWAT is abrogated by selective ablation of IL-33 or β1- and β2-AR in FRCs, or sympathetic denervation of iLNs, whereas replenishment of IL-33 reverses the impaired cold-induced beiging in iLN-deficient mice. Taken together, our study uncovers an unexpected role of FRCs in iLNs in mediating neuro-immune interaction to maintain energy homeostasis.

## Abstract (web summary)

Beiging and thermogenesis in white adipose tissue (WAT) is an important adaptive response to cold exposure, but how the brain senses cold and subsequently induces beiging remains unclear. Here, the authors show that sympathetic nerves stimulate lymph nodes to release IL-33, thereby mediating cold-induced beiging of WAT.

## Introduction

Adipose tissue (AT), which is compartmentalized into individual depots and distributed throughout the body, is a highly dynamic organ that plays important roles in the regulation of energy homeostasis, glucose and lipid metabolism, and immune responses. AT is functionally classified into white adipose tissue (WAT) for storage of excess energy as triglycerides and brown adipose tissue (BAT) for energy dissipation into heat. Additionally, brown-like beige adipocytes, which are scattered within WAT, possess similar thermogenic functions with brown adipocytes once being activated/recruited by environmental cold exposure or other thermogenic stimuli. Apart from their thermogenic properties, brown and beige adipocytes function as a metabolic sink for glucose and lipids. A growing body of evidence from both animal and human studies suggests that activation of brown/beige adipocytes represents a promising therapeutic strategy for obesity and its related cardiometabolic complications^1–3^.

Although classical brown adipocytes and inducible beige adipocytes are functionally similar, they arise from different progenitor cells and are activated by distinct mechanisms. Whilst classical brown adipocytes are derived from the myogenic lineage marker positive (Myf5^+^) lineage cells, beige adipocytes arise from Myf5^-^ progenitor cells. Cold exposure-induced activation of classical brown adipocytes in BAT is predominantly mediated by norepinephrine (NE) released from sympathetic nerve terminals whereas cold-induced biogenesis of beige adipocytes is dependent on sympathetic nervous system (SNS) as well as local microenvironment within WAT^4–6^. WAT is a highly heterogeneous organ consisting of adipocytes, various types of immune cells, stromal cells, blood vessels and nerve fibers^7^. Recent studies have identified numerous adipocyte-secreted adipokines and bioactive metabolites as important mediators of cold-induced beiging and thermogenesis of WAT through their paracrine or autocrine action^8–10^. In particular, type 2 innate lymphoid cells (ILC2s), which reside in subcutaneous WAT (scWAT) of both mice and humans, can be activated by IL-33^11^ and promote the biogenesis of beige adipocytes by secreting several type 2 cytokines, such as interleukin-5 (IL-5) and interleukin-13 (IL-13), which in turn facilitate the recruitment of eosinophils and polarization of M2 macrophages for beiging^11,12^. Furthermore, ILC2s secrete methionine-enkephalin peptide (MetEnk), which promotes beige adipogenesis by inducing UCP1 expression in WAT^11^. However, it remains unclear how sympathetic nerve system (SNS) and local immune microenvironment coordinate beiging and thermogenesis of WAT in response to cold challenge.

Cold-induced beiging of WAT displays obvious depot-specific differences. Beige adipocytes are readily detectable in scWAT even in ambient temperature, while visceral WAT (such as epididymal WAT, eWAT) is highly resistant to the formation of beige adipocytes^13^. These two adipose depots exhibit distinct patterns of sympathetic innervation as visualized by three-dimensional (3D) imaging^14^. Moreover, intra-depot variations within scWAT in biogenesis of beige adipocytes have been observed in several studies^14,15^. Cold-induced beiging occurs in the core region of scWAT, which is close to the inguinal lymph node (iLN) and highly vascularized compared with the remote areas^15^. A 3D imaging analysis of the entire scWAT with UCP1 immunofluorescence staining reveals the spread of beiging from the inguinal region (proximity to the iLN) to the dorsolumbar region (remote from the iLN) as the duration of cold exposure extends^14^. However, the molecular basis underlying the regional heterogeneity in beige biogenesis remains to be resolved.

Lymph nodes (LNs), a key component of the adaptive immune system distributed throughout the body, are anatomically and functionally associated with AT in both rodents and humans^16,17^. Almost all LNs are embedded in AT and most peripheral adipose depots contain one or more LNs. Adipocytes residing close to LNs are more active in lipolysis in response to local immune assault^18–21^, and are more reactive to NE than the distal adipocytes^22^. It has been suggested that LNs-surrounding adipocytes serve as energy reservoirs for the immediate and effective immune response in LNs^23,24^. In obesity, hypertrophic adipocytes disrupt both structure and function of LNs through elevated production of free fatty acids and reactive oxygen species that induce lymphocyte apoptosis^25–27^. Notably, the anatomical locations of LNs largely overlap with the regions of WAT that are susceptible to beiging in both rodents and humans, whereas the role of LNs in beiging of WAT has never been explored.

In this study, by using surgical removal and pharmacological depletion of LNs in mouse models, we identified inguinal LNs (iLNs) as an obligatory player of cold-induced beiging and thermogenesis of scWAT by mediating the crosstalk between SNS and type 2 immunity. Furthermore, we uncovered the underlying mechanism whereby sympathetic innervation of iLNs triggers the release of IL-33 from iLN-resident fibroblastic reticular cells (FRCs), which in turn activates ILC2s and downstream effectors to promote beiging of scWAT.

## Results

### Cold-induced biogenesis of beige adipocytes mainly aggregates in iLN-surrounding scWAT

**Fig. 1:**
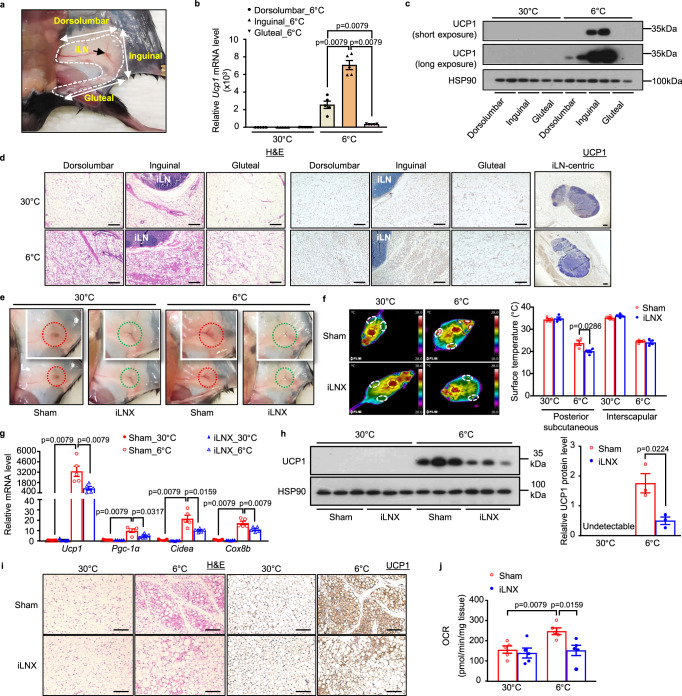
iLN promotes cold-induced beiging of scWAT. Eight-week-old male C57BL/6 N mice were housed at thermoneutral environment (30 °C) for 3 weeks and then subjected to exposure at 6 °C or 30 °C for 2 days. **a** In situ orientation of posterior scWAT (white dashed lines). iLN (black arrow); dorsolumbar, inguinal and gluteal regions (two-headed white arrows). **b**, **c** The mRNA (**b**) (*n* = 5) and protein (**c**) (*n* = 3) expressions of UCP1 in different regions of scWAT. **d** Representative images of different regions of scWAT stained with H&E (left) and immunohistochemical (IHC) staining for UCP1 (right). Scale bar, 200 μm. **e**–**j** Mice were subjected to bilateral inguinal lymphadenectomy (iLNX) or sham operation, and then housed at different temperatures as described above. **e** Macroscopic appearance of scWAT with (red-dotted circle) or without embedded-iLN (green-dotted circle) in sham-operated and iLNX mice. **f** Representative infrared images of sham-operated and iLNX mice housed at 30 °C or 6 °C. Quantification of the average surface temperature in the posterior subcutaneous (white-dotted circles) and interscapular regions (yellow-dotted circle) (right) (*n* = 4). **g**, **h** The mRNA level of thermogenic genes (**g**) (*n* = 5) and UCP1 protein expression (**h**) (*n* = 3) in scWAT. The right panel in (**h**) is the densitometric analysis for the relative abundance of UCP1 normalized with HSP90. **i** Representative images of H&E (left) and IHC staining for UCP1 (right) in scWAT. Scale bar, 200 μm. **j** Basal oxygen consumption rate (OCR) in the explants of scWAT (*n* = 5). All samples are biologically independent replicates. Data are presented as mean ± SEM. Statistical data were assessed using Mann–Whitney *U* test (**b**, **f**, **g**, **j**) or unpaired two-tailed Student’s *t* test (**h**). All the *p* values were two-sided. Source data are available as a Source Data file. kDa, relative molecular weight in kilodalton. See also Fig. S1–S2.

To explore whether the regional variation of beiging is related to the location of iLN, we compared the beiging capacity of scWAT in three different regions, *i.e*. inguinal region surrounding iLN, dorsolumbar and gluteal regions distal to iLN (Fig. 1a and fig. S1a). Interestingly, UCP1 mRNA and protein expressions from inguinal scWAT displayed the highest expression levels compared to the other two regions (Fig. 1b, c). UCP1 was only detectable in inguinal and dorsolumbar scWAT after long exposure time, but not in gluteal scWAT (Fig. 1c). To consolidate this finding, we also performed histological and immunohistological analyses, which showed the highest amount of multilocular UCP1^+^ beige adipocytes in the inguinal region of scWAT close to iLN compared with both dorsolumbar and gluteal regions after 2 days of cold exposure (Fig. 1d). These data collectively suggest that scWAT close to iLN is more prone to cold-induced beiging than other distal regions.

### Inguinal lymphadenectomy (iLNX) attenuates cold-induced formation of beige adipocytes in scWAT

To investigate the role of iLN in cold-induced beiging of scWAT, C57BL/6 N mice were subjected to iLNX or sham operation, followed by subjection to cold exposure (6 °C) or thermoneutral condition (30 °C) for 2 days after 3-week post-operative recovery (fig. S1b). The absence of iLN in the scWAT of iLNX group was confirmed by macroscopic examination (Fig. 1e) and staining with Evans Blue for detection of LNs (fig. S1c).

Both sham-operated and iLNX mice showed similar body temperatures when exposed to 6 °C for 48 h (fig. S1d). Notably, the dorsal skin temperature in the scWAT region of iLNX mice was significantly lower than that in sham-operated mice housed at 6 °C, whereas there was no difference in the interscapular BAT region (Fig. 1f), suggesting that removal of iLNs selectively impairs cold-induced adaptive thermogenesis in scWAT, but not in BAT. Indeed, the loss of iLN significantly inhibited cold-induced mRNA and protein expressions of UCP1, and markedly damped several other thermogenesis-related genes in scWAT, including *Pgc-1α*, *Cidea* and *Cox8b* (Fig. 1g, h). Histological examination of the inguinal scWAT revealed a profound morphological change towards clusters of multilocular UCP1^+^ adipocytes in sham-operated mice after cold challenge for 2 days, whereas this change was greatly diminished in iLNX mice (Fig. 1i). In parallel, the oxygen consumption rate (OCR) of scWAT explants from iLNX mice was significantly reduced when compared to the sham-operated mice under cold exposure (Fig. 1j). As expected, iLNX mice exhibited no significant changes of UCP1 expression or histological structures in BAT and eWAT under either 30 °C or 6 °C when compared with the sham-operated mice (fig. S1e–g).

We also investigated whether iLN is involved in acute cold exposure-induced beiging of scWAT. Time course analysis showed that UCP1 protein level was barely detectable in scWAT after 8-hour cold exposure, but was obviously increased after 16-hour cold exposure (Fig. S1h). Likewise, iLN removal led to  an attenuation of acute cold exposure (16 h)-induced beiging of scWAT, as evidenced by significantly reduced UCP1^+^ beige adipocytes and lower UCP1 protein level in scWAT when compared to sham-operated mice (Fig. S1i, j).

To further validate the role of iLN in cold-induced beiging of scWAT, we also performed unilateral iLNX in mice and used the contralateral side as an internal control (fig. S2a). Consistent with the findings in bilateral iLNX mice, scWAT with unilateral iLNX also exhibited significant reductions in local temperature, induction of thermogenic gene expression and OCR upon cold exposure, when compared to its contralateral sham control (fig. S2b–e). Taken together, these data demonstrate that the removal of iLN selectively impairs cold-mediated beiging in neighbouring scWAT.

### Cold-induced activation of ILC2s and commitment of beige cell lineage are impaired in iLNX mice

**Fig. 2:**
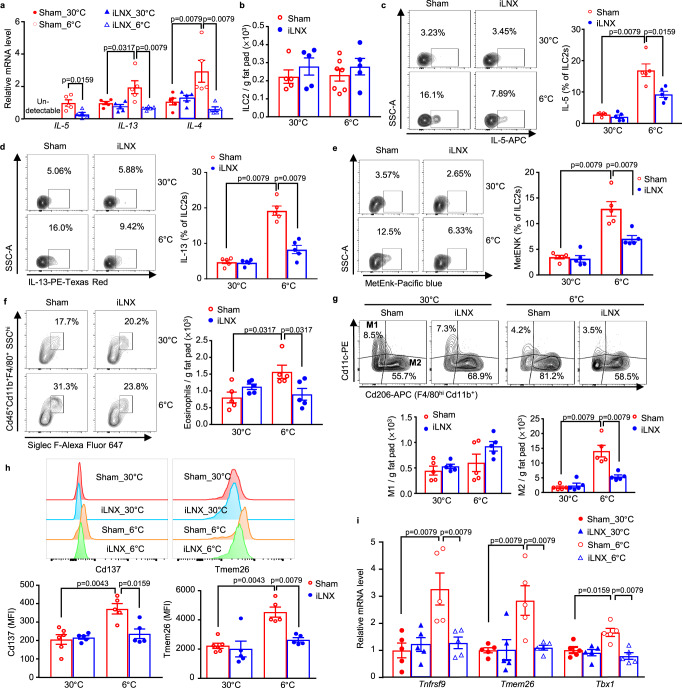
iLN regulates cold-induced activation of ILC2s, recruitment of eosinophils, polarization of M2 macrophages and beiging of adipocytes in scWAT. SVF was isolated from inguinal scWAT of 11-week-old iLNX or sham-operated male C57BL/6 N mice after housing at 30 °C for 3 weeks and subsequently subjected to 30 °C or 6 °C for 2 days. **a** The mRNA expression of type 2 cytokines (*n* = 5). **b** The absolute number of ILC2s (Lin^-^Cd5^-^Cd45^+^Cd127^+^IL33R^+^) in entire scWAT. For Sham_30 °C, iLNX_30 °C and iLNX_6 °C (*n* = 5) or Sham_6 °C (*n* = 7). **c–e** Representative contour plots for ILC2s (left) and quantification of the percentage (right) of IL-5- (**c**) IL-13- (**d**) and MetENK- (**e**) positive cells in activated ILC2s from scWAT (*n* = 5). **f** Representative contour plots for eosinophils (CD45^+^CD11b^+^F4/80^+^SiglecF^+^SCC^hi^) (left) and quantification of the absolute number of eosinophils in entire scWAT (right) (*n* = 5). **g** Representative contour plots from flow cytometry analysis for macrophages (M1, F4/80^+^Cd11b^+^Cd11c^+^; M2, F4/80^+^Cd11b^+^Cd206^+^) (top) and quantifications of the absolute number of M1 and M2 macrophages in entire scWAT (bottom) (*n* = 5). **h** Representative histogram overlays of flow cytometric analysis for beige-specific markers (top) and quantification of median fluorescence intensity (MFI) of Cd137 and TMEM26 (bottom) in adipocyte progenitors. For Sham_30 °C (*n* = 6) or iLNX_30 °C, Sham _6 °C and iLNX_6 °C (*n* = 5). **i** Real-time PCR analysis of the mRNA expression of beige progenitor markers (*n* = 5). All samples are biologically independent replicates. Data are presented as mean ± SEM. All statistical data were assessed using the Mann–Whitney *U* test. All the *p* values were two-sided. Source data are available as a Source Data file. See also Fig. S2–S6.

Since the sham-operated and iLNX mice show comparable functional BAT, we hypothesized that iLN may regulate the local microenvironment of scWAT required for cold-induced biogenesis of beige adipocytes^8,11,28^. In line with previous reports^8,12^, cold exposure significantly induced the mRNA expression of several type 2 cytokines (*IL-5*, *IL-13* and *IL-4*) in scWAT of sham-operated mice (Fig. 2a), whereas cold-induced elevation of these type 2 cytokines in scWAT was largely abolished by surgical removal of iLNs. As ILC2s in WAT promote beige fat biogenesis by secreting type 2 cytokines and MetEnk^11,12^, we next examined the abundance of ILC2s (Lin^-^Cd5^-^Cd45^+^Cd127^+^IL33R^+^) in scWAT with flow cytometric analysis (fig. S3a). Consistent with a previous study^9^, the number of ILC2s in scWAT was comparable between the two groups of mice housed under 30 °C or 6 °C (Fig. 2b, fig. S4a). In sham-operated mice, cold exposure increased the expression of IL-5, IL-13 and MetEnk in scWAT-resident ILC2s, especially in the inguinal region (Fig. 2c–e, and fig. S4b, c). However, these cold-induced changes were largely abrogated in iLNX mice (Fig. 2c–e). Notably, surgical depletion of iLN had no obvious effect on the density of lymphatic and blood vessels, as well as in the recruitment of γδT cells, neutrophils and dendritic cells in iLN-surrounding scWAT (fig. S5a, b and fig. S6a–f). In addition, unilateral iLNX had no impact on the activation of ILC2s and norepinephrine content in the contralateral sham scWAT (fig. S2f–h). Consistently, cold exposure-induced increases in the abundance of eosinophils (F4/80^+^Cd11b^+^Siglec F^+^) and M2 macrophages (F4/80^hi^Cd11b^+^Cd206^+^), both of which are the downstream effectors of ILC2s that mediate beiging of scWAT^12,28^, were profoundly compromised by the removal of iLN (fig. S3b, c, and Fig. 2f, g). Conversely, there was no significant difference in the percentage of M1 macrophages in scWAT between sham-operated and iLNX groups under 30 °C or 6 °C (Fig. 2g). Taken together, these results suggest that iLN is required for activation of ILC2s and recruitment of eosinophils/M2 macrophages during cold-induced beiging of scWAT.

Cold exposure induces biogenesis of beige adipocytes by promoting the commitment of bipotential adipocyte precursors into beige adipocyte lineage^29^. Therefore, we next investigated the impacts of iLN depletion on modulating the lineage commitment to beige adipocyte precursors (Lin^-^Cd45^-^Cd31^-^Sca1^+^Pdgfrα^+^Tmem26^+^ or Cd137^+^) using flow cytometry analysis (fig. S3d). The abundance of beige adipocyte precursors in SVFs from scWAT was significantly enhanced by cold challenge in sham-operated mice, whereas such an effect of cold challenge was obviously attenuated in iLNX mice (Fig. 2h). Likewise, cold-induced gene expression of several beige lineage markers, including *Tnfrsf9 / Cd137, Tmem26 and Tbx1*, was abrogated by the loss of iLN (Fig. 2i). In addition, a subset of adipocyte precursors (Lin^-^Cd45^-^Cd31^-^Sca1^+^CD81^+^) (fig. S3e) that only gives rise to beige adipocytes in scWAT^30^ also showed a significant increase of cold-induced commitment of beige-specific adipocyte precursors in SVFs from scWAT, but such induction was attenuated in iLNX mice (fig. S1k). These results, together with the finding that iLN mediates the activity of ILC2s (Fig. 2c–e), support the notion that iLN regulates type 2 immunity and eventually leads to cold-induced biogenesis of beige adipocytes.

### iLN FRC-derived IL-33 is indispensable for cold-induced activation of ILC2s and beiging of scWAT

**Fig. 3:**
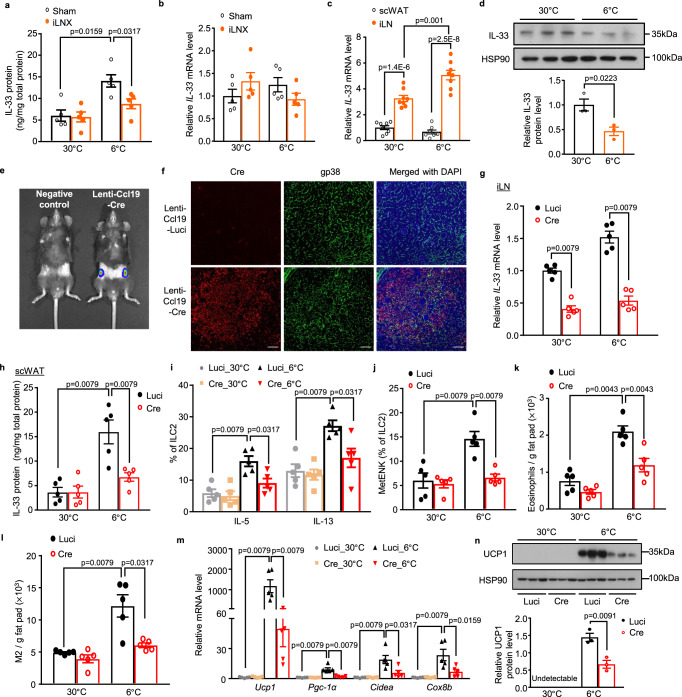
iLN FRC-derived IL-33 is indispensable for cold-induced activation of ILC2s and beiging of scWAT. **a–d** Sham-operated and iLNX male C57BL/6 N mice were exposed to 6 °C or 30 °C for 2 days. **a**, **b** ELISA analysis of protein level (a) and the mRNA expression of IL-33 (**b**) in scWAT of sham-operated and iLNX mice (*n* = 5). **c** The mRNA level of IL-33 in scWAT and iLN (*n* = 8). **d** IL-33 protein level in iLN determined by Western blot (top) and densitometric quantification of IL-33 (bottom) (*n* = 3). **e**–**n** Lentivirus encoding FLAG-tagged Cre and luciferase (Lenti-Ccl19-Cre) or luciferase only (Lenti-Ccl19-Luci) driven by the Ccl19 promoter was directly injected into iLNs (7.5 × 10^6^ Transduction Units [TU] per side) of eight-week-old male IL33^fl/fl^ mice. Seven days after lentiviral injection, mice were housed at 30 °C for 3 weeks followed by 2-day cold exposure (6 °C) or continued to be housed at 30 °C for another 2 days. **e** IVIS Lumina imaging analysis showing luminescence intensity in mice with (Lenti-Ccl19-Cre) or without lentivirus injection (negative control) after intraperitoneal injection of luciferin (150 mg/kg) for 10 min. **f** Immunofluorescent staining with primary antibodies against Cre recombinase or gp38 (a cell surface marker of FRCs) in iLN section; Scale bar, 50 μm **g** The mRNA level of IL-33 in iLN (*n* = 5). **h** ELISA analysis for IL-33 protein level in scWAT (*n* = 5). **i**, **j** Quantification of IL-5, IL-13 (**i**) and MetENK (**j**) in ILC2s using flow cytometric analysis (*n* = 5). **k**, **l** Flow cytometric analysis of absolute numbers of eosinophils (**k**) and M2 macrophages (**l**) (*n* = 5). **m** The mRNA expression of thermogenic genes in scWAT, determined by real-time PCR (*n* = 5). **n** Western blot analysis for UCP1 protein expression in scWAT (top) and densitometric quantification of UCP1 (bottom) (*n* = 3). All samples are biologically independent replicates. Data are presented as mean ± SEM. Statistical data were assessed using unpaired two-tailed Student’s test (**c**, **d, n**) or Mann–Whitney *U* test **(a**, **g**–**m)**. All the *p* values were two-sided. Source data are available as a Source Data file. kDa, relative molecular weight in kilodalton. See also Fig. S2, S7–S11.

IL-33 is an activator of ILC2s in the biogenesis of beige adipocytes^11,12^. We found that cold exposure could significantly increase IL-33 protein level in scWAT, especially in the inguinal region (Fig. 3a and fig. S4d). However, the cold-induced elevation of IL-33 protein level in scWAT was significantly attenuated in iLNX mice (Fig. 3a), whereas the mRNA level of *IL-33* remained unchanged under either 30 °C or 6 °C (Fig. 3b). Likewise, a significant decrease was only observed in cold-induced elevation of IL-33 protein in scWAT with the unilateral iLNX, but not in the contralateral sham side (fig. S2i, j). Interestingly, the mRNA level of *IL-33* in iLN was 3-fold higher than scWAT and cold induction further upregulated its mRNA level in iLN but not in scWAT (Fig. 3c). In contrast, the protein abundance of IL-33 in iLN was significantly reduced (Fig. 3d and fig. S7a), whereas IL-33 protein level in the non-permeabilized scWAT sections was increased in response to cold exposure (fig. S7b). Further flow cytometry analysis showed that IL-33 was mainly expressed in the fibroblastic reticular cells (FRCs) (defined as Cd45^-^Cd31^-^gp38^+^ cells) in iLN (fig. S7c–d)^31,32^ and the relative percentage of FRCs was unaffected after cold challenge (fig. S7e). Although lymphatic endothelial cells (LECs) in iLNs have been reported to express IL-33^33^, our results revealed that the expression level of IL-33 in FRCs was approximately 3-fold higher than in LECs (defined as Cd45^-^Cd31^+^gp38^+^ cells) (fig. S7d). Furthermore, a significant decrease in intracellular IL-33 level was observed only in FRCs but not LECs after 2-day cold exposure when compared to the mice housed in a thermoneutral condition (fig. S7f). Immunofluorescence staining of IL-33 and lymphatic vessel endothelial receptor 1 (LYVE-1, the marker of lymphatic vessels) demonstrated an obvious co-localization between IL-33 and LYVE-1 in iLN after cold challenge, indicating that IL-33 released from FRCs could enter lymphatic vessels (fig. S8a). Consistently, intranodally injected FITC-conjugated dextran could be detected in lymphatic vessels within iLN after 2 minutes, and was then migrated from iLN to its surrounding inguinal scWAT, which was partially co-localized with perilipin after injection for 20 min (fig. S8b, c). Taken together, these findings suggest that FRCs in iLN are an important source of IL-33, which is released to its adjacent scWAT possibly via lymphatic vessels to exert its functions during cold exposure.

Given that endogenous IL-33 is abundantly expressed in the FRCs, we next investigated whether FRC is a major contributor to the elevated IL-33 protein in scWAT during cold challenge by specific ablation of IL-33 in iLN FRCs using the Ccl19 promoter, which is specifically expressed in FRCs^32^. To this end, lentivirus carrying FLAG-tagged Cre recombinase and luciferase driven by a Ccl19 promoter (Lenti-Ccl19-Cre) was directly injected into the iLN of IL-33^fl/fl^ mice^34^. In vivo imaging analysis for luminescence intensity showed the luciferase expression predominantly in iLN but not in other organs (Fig. 3e). Furthermore, the Ccl19 promoter-driven expression of FLAG-tagged Cre was detected predominantly in iLN FRCs but not in scWAT, as determined by co-staining with the FRC marker gp38 and adipocyte marker perilipin (Fig. 3f and fig. S9a). Consistently, flow cytometric analysis showed that approximately 80% of FRCs isolated from iLN of mice with Lenti-Ccl19-Cre injection were Cre^+^, whereas Cre expression was virtually undetectable in the control group (fig. S9c). We also performed a flow cytometric analysis to exclude the possible expression of Cre recombinase in dendritic cells in iLN^34^. Our results showed undetectable Cre expression in dendritic cells (CD45^+^CD11b^+^CD64^-^CD11c^+^) isolated from iLN of mice with Lenti-Ccl19-Cre injection (fig. S9b).

The mRNA expression of *IL-33* in FRCs sorted from iLNs was markedly decreased by approximately 65% in IL-33^fl/fl^ mice injected with Lenti-Ccl19-Cre when compared with the mice injected with lentivirus expressing the luciferase control (Fig. 3g). Consistently, flow cytometric analysis also showed a significant reduction of intracellular IL-33 protein expression in Cre-expressing FRCs when compared to the control group (fig. S9d), thus confirming the efficient and specific deletion of IL-33 in FRCs of iLNs. Notably, specific ablation of IL-33 in iLN FRCs led to significant reductions in cold-induced elevation of IL-33 protein in scWAT, activation of ILC2s, recruitment of eosinophils and M2 macrophages, as well as upregulation of the thermogenic genes (Fig. 3h–n). Lentivirus-mediated depletion of IL-33 did not influence the density of blood vessels, absolute number of ILC2s, M1 macrophages, γδT cells, neutrophils and dendritic cells as well as the sympathetic innervation of scWAT, as determined by immunofluorescence staining of CD31 (the marker of blood vessels), flow cytometric analysis of the relevant immune cells, norepinephrine content and TH protein level (fig. S10a–h). Unilateral injection of Lenti-Ccl19-Cre also had no impact on the contralateral sham iLN and scWAT with respect to IL-33 protein levels, ILC2 activation and thermogenic gene expression (fig. S11a–c). Taken together, these findings support FRCs in iLN as a major source of IL-33 that drive ILC2 activation and subsequent beiging in the local scWAT.

**Fig. 4:**
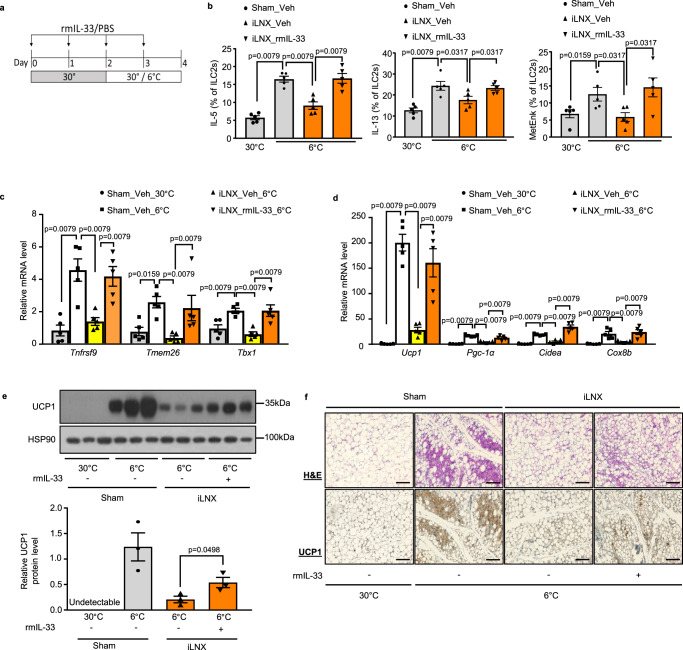
Replenishment of IL-33 rescues the defective cold-induced beiging in iLN-depleted mice. **a**–**f** Eight-week-old male C57BL/6 N mice after bilateral iLNX or sham operation were housed at 30 °C for three weeks. 250 ng recombinant mouse IL-33 protein (rmIL-33) or PBS (Veh) was directly injected into the scWAT for 4 consecutive days. Mice were then subjected to 6 °C or continued to be housed at 30 °C for 2 days after the first two injections. **a** Schematic diagram showing the experimental design. **b** Quantification of the percentage of IL-5-, IL-13- and MetENK-positive cells in activated-ILC2s from scWAT using flow cytometric analysis (*n* = 5). **c**, **d** The mRNA expression of beige-specific markers (**c**) and thermogenic genes (**d**) in scWAT (*n* = 5). **e** Western blot analysis for UCP1 protein expression in scWAT (top) and densitometric quantification for the relative abundance of UCP1 normalized with HSP90 (bottom) (*n* = 3). **f** Representative images of H&E and IHC staining for UCP1 in scWAT. Scale bar, 200 μm. All samples are biologically independent replicates. Data are presented as mean ± SEM. Statistical data were assessed using Mann–Whitney *U* test (**b–d**) or unpaired two-tailed Student’s *t* test (**e**). All the p values were two-sided. Source data are available as a Source Data file. kDa, relative molecular weight in kilodalton. See also Fig. S12–S13.

As the cold-induced elevation of IL-33 protein level in scWAT is abrogated by surgical removal of iLN, we next asked whether supplementation of recombinant mouse IL-33 (rmIL-33) into iLNX mice could restore the ability of cold-induced beiging in scWAT (Fig. 4a). Administration of rmIL-33 in the local scWAT fat pads of iLNX mice led to a marked restoration in expressions of IL-5, IL-13 and MetEnk, as determined by flow cytometric analysis (Fig. 4b), real-time PCR analysis of beige lineage and thermogenic gene markers (Fig. 4c, d) and protein expression of UCP1 (Fig. 4e). Similarly, administration of rmIL-33 only had a local effect on ILC2 activation and UCP1 expression in the injected side of scWAT with unilateral iLNX, but had no systemic effect on the contralateral sham control side without rmIL-33 injection (fig. S12a–e). Consistent with previous reports^9,11,12,35^, the abundance of ILC2s was increased significantly upon receiving the injection of rmIL-33, but not in the groups which were only exposed to cold environment (fig. S13a). Likewise, histological examination of scWAT revealed more multilocular UCP1^+^ adipocytes after the injection of rmIL-33 in iLNX mice under 6 °C (Fig. 4f), whereas treatment with rmIL-33 had no obvious effect on UCP1 expression in BAT and histological structure of eWAT (fig. S13b, c). Taken together, these observations suggest that supplementation of rmIL-33 reverses the impairment of cold-induced biogenesis of beige adipocytes in iLNX mice, highlighting the importance of the iLN-IL-33-ILC2 axis in beige biogenesis.

### Cold exposure enhances sympathetic innervation of iLN and activates β1- and β2-adrenergic receptor signaling in FRCs to promote the release of IL-33

**Fig. 5:**
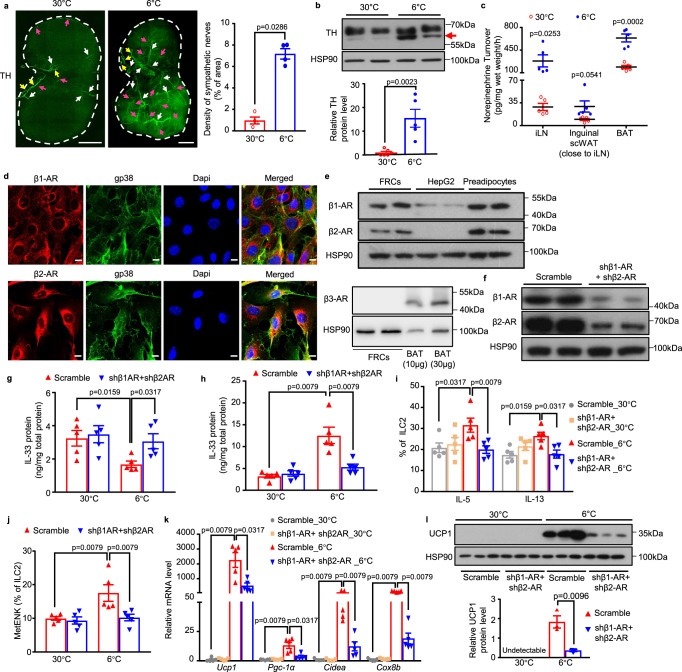
Cold exposure enhances sympathetic innervation of iLN and activates β1- and β2-AR signaling in FRCs to promote the release of IL-33. **a**–**d** Eight-week-old male C57BL/6 N mice were housed at 30 °C for three weeks before subjecting to exposure at 6 °C or 30 °C for 2 days. **a** Whole-mount imaging of tyrosine hydroxylase (TH, green) in iLNs (left). Arrowheads indicate distinct patterns of sympathetic innervation: (1) white arrows: along blood vessels; (2) yellow arrows: nerve bundles; (3) magenta arrows: neurite projections. Scale bar, 500 μm. The quantification of TH-positive signal using ImageJ (right) (*n* = 4). **b** Western blot analysis for TH in iLN (top) and densitometric quantification of TH protein (bottom) (*n* = 6 for 30 °C and *n* = 5 for 6 °C are from one experiment and processed in parallel). **c** Norepinephrine turnover (NETO) was determined by LC/MS analysis (*n* = 5). **d** Representative confocal images of cultured FRCs stained with antibodies against adrenergic receptors (β1-AR or β2-AR) and gp38; Scale bar, 10 μm. **e** Protein expression of β1-AR, β2-AR and β3-AR in FRCs. HepG2, SVF-derived preadipocytes and BAT were used as controls (*n* = 2). **f**–**l** Lentiviruses encoding shRNA against β1-AR or β2-AR driven by the Ccl19 promoter (5 × 10^6^ TU for each) were co-injected into iLNs of eight-week-old male C57BL/6 N mice. Lentivirus encoding scrambled shRNA under the control of Ccl19 promoter (1 × 10^7^ TU) was used as control. Seven days after injection, mice were housed at 30 °C for 3 weeks followed by cold exposure at 6 °C or 30 °C for 2 days. **f** Western blot analysis for β1-AR and β2-AR protein expressions in FRCs sorted from iLNs (*n* = 2). **g**–**h** ELISA analysis for IL-33 protein level in iLN (**g**), scWAT (**h**) (*n* = 5). **i**, **j** Quantification for IL-5, IL-13 (**i**), and MetENK (**j**) in ILC2s using flow cytometric analysis (*n* = 5). **k** The mRNA expression of thermogenic genes in scWAT (*n* = 5). **l** Western blot analysis for UCP1 protein expression in scWAT (top) and densitometric quantification of UCP1 protein (bottom) (*n* = 3). All samples are biologically independent replicates. Data are presented as mean ± SEM. Statistical data were assessed using Mann–Whitney *U* test (**a**, **g**–**k**) or unpaired two-tailed Student’s *t* test (**b**, **c, l**). All the *p* values were two-sided. Source data are available as a Source Data file. kDa, relative molecular weight in kilodalton. See also Fig. S14-S17.

Previous studies have shown that LNs are innervated by SNS in both mice and humans^36,37^. Therefore, we next investigated whether cold exposure modulates SNS activity within iLN. Intriguingly, the density of tyrosine hydroxylase-positive (TH^+^) sympathetic nerves was obviously increased (~7-fold) after cold exposure when compared to the mice housed in a thermoneutral condition, as determined by both whole-mount immunostaining and confocal imaging analysis of iLNs (Fig. 5a and fig. S14a, b). Consistently, TH protein level within iLN was also markedly induced upon cold exposure for 2 days (Fig. 5b). Although TH expression has been reported to be detectable in the lymphocytes, our immunofluorescence staining revealed no colocalization between TH and Cd3^+^ lymphocytes in iLN (fig. S14c). Consistently, the sympathetic activity was markedly enhanced in iLN (~10-fold) upon cold stimulation, as determined by norepinephrine turnover (NETO) (Fig. 5c).

Considering the abundant expression of IL-33 in FRCs, we further hypothesized that sympathetic outflow to iLN may promote the synthesis and release of IL-33 from FRCs in iLN to the surrounding scWAT. Therefore, we isolated FRCs from mouse iLN (fig. S15a, b) to interrogate whether FRCs express adrenergic receptors. Both immunofluorescence staining and immunoblotting analyses revealed the presence of β1- and β2-adrenergic receptors (β-ARs) but not β3-AR in gp38^+^ FRCs (Fig. 5d, e). Furthermore, treatment of FRCs with the β-ARs agonist isoproterenol significantly induced the release of IL-33 into the extracellular medium but this effect was partially blocked by the treatment of PKA inhibitor H89 (fig. S15c). Likewise, the protein kinase A (PKA) activator, forskolin partially mimicked the effects of the β-ARs agonist on the release of IL-33 in FRCs (fig. S15c), suggesting that catecholamines-mediated the activation of PKA may contribute to its induction of IL-33 expression. Interestingly, significant smaller spaces between the reticular network branches in iLN but unchanged FRC numbers were observed in mice after 2-day cold exposure (fig. S16a and fig. S7e), suggesting the “contraction” of FRC networks. Likewise, co-staining of F-actin (the marker of actomyosin cytoskeleton) and IL-33 showed that F-actin was mainly distributed along the cell border in untreated FRCs (fig. S16b), indicative of the absence of stress fiber for contraction^38,39^. Treatment with the β-ARs agonist isoproterenol led to a redistribution of F-actin throughout the cells, whereas this change was partially blocked by the PKA inhibitor H89 (fig. S16b). Moreover, IL-33 protein in the nucleus was obviously reduced by treatment with isoproterenol when compared to untreated cells (fig. S16b). In contrast, treatment with isoproterenol did not cause any elevation in lactate dehydrogenase (LDH) (fig. S15d), thus excluding the involvement of necrotic cells in the β-ARs agonist-induced release of IL-33. Taken together, these findings collectively suggest that adrenergic stimulation induces the release of IL-33 from FRCs possibly by PKA-mediated mechanical stretch.

To further explore the physiological roles of β1- and β2-AR signaling in FRCs, we co-injected lentiviruses encoding the Ccl19 promoter-driven shRNA against β1- or β2-AR into iLNs of C57BL/6 N wild-type mice. The knockdown efficiency of β1- and β2-AR was validated by western blot analysis showing ~75.8% and 83.9% reductions in β1- and β2-AR protein expressions respectively in sorted FRCs compared to those injected with lentivirus encoding scrambled control (Fig. 5f). Upon cold exposure, knockdown of β1- and β2-AR resulted in significantly increased IL-33 protein level in iLN (Fig. 5g) but reduced IL-33 level in scWAT (Fig. 5h), indicating impaired secretion of IL-33 from FRCs in iLN into its surrounding scWAT. Likewise, mice with selective knockdown of β1- and β2-AR in iLN FRCs exhibited obvious reductions in cold-induced ILC2 activation, recruitment of eosinophils and M2 macrophages, and defective beiging in scWAT (Fig. 5i–l, and fig. S17a, b), whereas vascularization and recruitment of ILC2s, M1 macrophages, γδT cells, neutrophils and dendritic cells remained unchanged in scWAT (fig. S17c–h). Collectively, these results suggest that cold-induced release of NE from sympathetic nerves acts on β1 and β2-AR-expressing FRCs to promote the synthesis and secretion of IL-33 from iLN to its surrounding scWAT.

### Sympathetic denervation of iLNs attenuates cold-induced beiging in scWAT

**Fig. 6:**
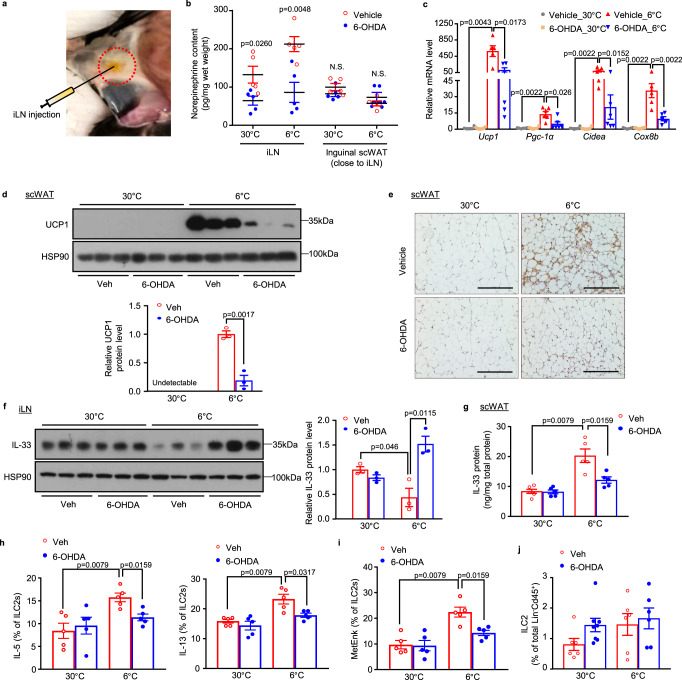
Sympathetic denervation of iLN compromises IL-33 release, dampens ILC2 activation and beiging of scWAT in response to cold challenge. Eight-week-old male C57BL/6 N mice were subjected to bilateral sympathetic denervation of iLN by local injection of 6-OHDA (9 mg/ml in 0.15 M NaCl) or 0.15 M NaCl (Veh) directly into iLNs. Afterwards, mice were housed at 30 °C for 3 weeks followed by cold exposure (6 °C) or continued to be housed at 30 °C for another 2 days. **a** A macroscopic image showing the injection site of iLN embedded in scWAT. **b** LC/MS-based measurement of norepinephrine content in iLN and its surrounding inguinal scWAT (*n* = 5). **c** The mRNA expression of thermogenic genes in scWAT (*n* = 6). **d** UCP1 protein level in scWAT (top) and densitometric analysis for the relative abundance of UCP1 normalized with HSP90 (bottom) (*n* = 3). **e** Representative images of IHC staining for UCP1 in scWAT. Scale bar, 200 μm. **f** IL-33 protein level in iLN (left) and densitometric analysis for the relative abundance of IL-33 normalized with HSP90 (right) (*n* = 3). **g** ELISA analysis of IL-33 protein level in scWAT (*n* = 5). **h**, **i** Quantification of the percentage of IL-5- and IL-13- (**h**), and MetENK- (**i**) positive cells in activated ILC2s using flow cytometric analysis (*n* = 5). **j** Flow cytometric analysis for the proportion of ILC2s in lineage negative leukocytes in SVF. For Veh_30 °C, Veh_6 °C and 6-OHDA_6 °C (*n* = 6) or 6-OHDA_30 °C (*n* = 8). All samples are biologically independent replicates. Data are presented as mean ± SEM. Statistical data were assessed using the Mann–Whitney *U* test **(c**, **g**–**i)** or unpaired two-tailed Student’s *t* test (**b**, **d**, **f**). All the *p* values were two-sided. Source data are available as a Source Data file. kDa, relative molecular weight in kilodalton; NS not significant. See also Fig. S18.

To investigate whether sympathetic innervation of iLNs plays a role in cold-induced beiging in iLN-surrounding scWAT, we conducted sympathetic denervation via direct injection of the catecholaminergic neurotoxin 6-hydroxydopamine (6-OHDA) into both sides of iLN (Fig. 6a). LC/MS analysis showed that cold exposure markedly increased NE levels in iLN injected with vehicle control, whereas such an effect of cold challenge on the elevation of NE was largely abrogated in iLN injected with 6-OHDA. On the other hand, injection of 6-OHDA did not affect the NE content in scWAT under either the thermoneutral or cold condition (Fig. 6b), confirming a selective and effective sympathetic denervation within iLN, without obvious disturbance of its surrounding scWAT. Notably, cold-induced beiging of scWAT was largely abrogated in iLN-denervated mice, as evidenced by dramatically decreased expression of thermogenic genes (*Ucp1*, *Pgc-1α*, *Cidea* and *Cox8b*) (Fig. 6c), reduced UCP1 protein level and the number of UCP1^+^ beige adipocytes compared to the vehicle group after cold exposure (Fig. 6d, e). Furthermore, the cold-induced release of IL-33 protein in iLN and the increase of IL-33 protein level in scWAT were largely attenuated in iLN-denervated mice (Fig. 6f, g). Such changes in iLN-denervated mice were accompanied by impaired ability of ILC2s to secrete IL-5, IL-13 and MetEnk in response to cold challenge (Fig. 6h, i), despite the unchanged abundance of ILC2s (Fig. 6j). Furthermore, unilateral injection of 6-OHDA into iLNs also led to significant decreases in cold-induced ILC2 activation and beiging in the scWAT with iLN denervation when compared to the contralateral sham control (fig. S18a–c), thus excluding the possible systemic effect of bilateral sympathetic denervation of iLN. Collectively, these results support the notion that sympathetic innervation of iLN modulates beiging of scWAT by controlling the release of IL-33.

### Pharmacological ablation of iLNs impairs the development of cold-inducible beige adipocytes in scWAT

**Fig. 7:**
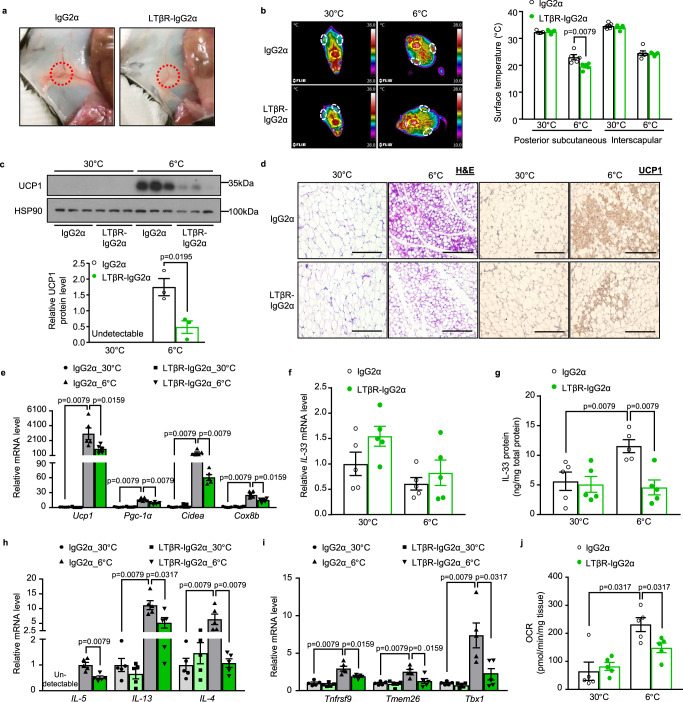
LTβR-IgG2α-induced depletion of iLNs impairs beiging of scWAT during cold exposure. Pregnant female C57BL/6 N mice were intravenously injected with lymphotoxin beta receptor-immunoglobulin G2α fusion protein (LTβR-IgG2α) (200 μg) on embryonic day 13 (E13) and E14 to generate iLN-depleted progeny mice. Mice receiving IgG2α isotype were used as controls. **a** Macroscopic photos showing the absence of iLN in LTβR-IgG2α-treated mice (red-dotted circle). **b** Representative infrared images of iLN-depleted mice and their controls housed at 30 °C or 6 °C (left), and the quantification of average surface temperature in the posterior subcutaneous (white-dotted circles) and interscapular regions (yellow-dotted circle) (right) (*n* = 5). **c** UCP1 protein expression in scWAT (top) and densitometric analysis for the relative abundance of UCP1 normalized with HSP90 (bottom) (*n* = 3). **d** Representative images of H&E (left) and IHC staining for UCP1 (right) in scWAT. Scale bar, 200 μm. **e** The mRNA levels of thermogenic genes in scWAT (*n* = 5). **f**, **g** The mRNA expression (**f**) and ELISA analysis of protein level (**g**) of IL-33 in scWAT (*n* = 5). **h**, **i** The mRNA expression of type 2 cytokines (**h**) and beige-specific markers (**i**) (*n* = 5). **j** Basal OCR in the explants of scWAT (*n* = 5). All samples are biologically independent replicates. Data are presented as mean ± SEM. Statistical data were assessed using the Mann–Whitney *U* test (**b**, **e**, **g**–**j**) or unpaired two-tailed Student’s *t* test (**c**). All the *p* values were two-sided. Source data are available as a Source Data file. kDa, relative molecular weight in kilodalton. See also Fig. S19–21.

Treatment of pregnant mice with lymphotoxin-β receptor-immunoglobulin fusion protein (LTβR-IgG2α), which acts as a soluble decoy for LTβR, has been shown to result in newborns without peripheral and peri-pancreatic LNs^40,41^. To further verify the role of iLN in cold-induced beiging of scWAT, we generated mice without peripheral LNs by intravenous injection of LTβR-IgG2α or IgG2α (as control) into female mice on E13 and E14 during gestation (fig. S19a). Successful ablation of iLN in the offspring of LTβR-IgG2α-treated mice was confirmed by ultrasonic imaging analysis (fig. S19b) and macroscopic examination (Fig. 7a). Consistent with a previous report^41^, histological structures of spleen and thymus remain intact with distinguishable red and white pulp regions (fig. S19c), indicating that LTβR signaling remains unaltered postnatally.

In line with the findings from iLNX mice, there was no obvious difference in rectal temperature between IgG2α-treated control and LTβR-IgG2α-treated iLN-free offspring under either 30 °C or 6 °C (fig. S19d). However, iLN-depleted mice in cold environment exhibited obviously reduced skin temperature in the inguinal region (Fig. 7b), markedly decreased UCP1 protein level, reduced formation of multilocular, UCP1^+^ adipocytes, and significantly downregulated expression of several thermogenic genes (*Ucp1*, *Pgc-1α*, *Cidea* and *Cox8b*) in scWAT compared to IgG2α-treated control littermates (Fig. 7c–e). Consistently, cold exposure increased protein, but not mRNA level of *IL-33*, and induced gene expression of several type 2 cytokines (*IL-5, IL-13 and IL-4*) and beige cell lineage markers (*Tnfrsf9, Tmem26 and Tbx1*) in IgG2α-treated offspring with iLN, whereas all these effects of cold exposure were largely compromised in LTβR-IgG2α-treated iLN-free offspring (Fig. 7f–i). Furthermore, OCR of scWAT explants from LTβR-IgG2α-treated iLN-free offspring was much lower than that in IgG2α-treated offspring under cold environment (Fig. 7j). Considering that LTbR-IgG2α treatment has also been shown to deplete axillary and popliteal lymph nodes of offspring mice^42^, we next investigated the impact of depletion of axillary and popliteal lymph nodes on cold-induced beiging of their surrounding adipose tissues. In line with previous reports^43,44^, cold-induced beiging was hardly detectable in white adipose tissue surrounding popliteal lymph nodes, but was obviously present in those surrounding axillary lymph nodes, whereas such cold-induced beiging was largely abrogated in mice with depletion of axillary lymph nodes (fig. S20a–c).

Similar to the observations in mice with surgical removal of iLN, pharmacological depletion of iLN did not affect the density of lymphatic and blood vessels (fig. S21a, b), or the abundance of ILC2s, M1 macrophages, γδT cells, neutrophils and dendritic cells in iLN-surrounding scWAT, despite a significant attenuation in cold-induced recruitment of eosinophils and M2 macrophages (fig. S21c–i). As expected, the absence of iLN by treating pregnant female mice with LTβR-IgG2α did not affect UCP1 expression or histological structures of BAT and eWAT under either 30 °C or 6 °C (fig. S19e–g).

## Discussion

**Fig. 8:**
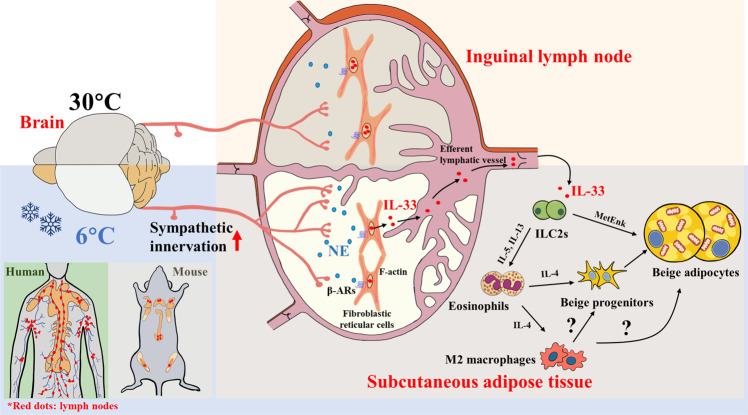
iLN couples sympathetic signaling and type 2 immune responses to mediate cold-induced beiging of scWAT by releasing IL-33 from FRCs. Cold exposure enhances sympathetic innervation of iLN, thereby releasing norepinephrine (NE) to act on beta adrenergic receptors (β-ARs) in fibroblastic reticular cells (FRCs). Adrenergic stimulation induces mechanical response (contraction) of FRCs, thereby releasing IL-33 into iLN-surrounding scWAT through the efferent lymphatic vessel, and subsequently activates group 2 innate lymphoid cells (ILC2s) and their downstream type 2 immune responses for the biogenesis of beige adipocytes. Note that it is still debatable on whether and how M2 macrophages regulate cold-induced beiging in subcutaneous WAT.

Although it is well-established that SNS mediates cold- and diet-induced adaptive thermogenesis in BAT through direct actions on β-ARs in brown adipocytes by releasing NE, the mechanism whereby SNS promotes biogenesis and thermogenesis of beige adipocytes in WAT remains obscure. Unlike classical brown adipocytes which are constitutively present, beige adipocytes are barely detectable at room temperature and require de novo biogenesis or transdifferentiation upon cold exposure. In the current study, we identified a previously unappreciated pathway through which cold-induced sympathetic outflow to iLN facilitates the release of IL-33 from FRCs, thereby creating an immune microenvironment favorable for the biogenesis of beige adipocytes (Fig. 8). These findings reveal FRCs as a major source of IL-33 for cold-induced beiging of scWAT, uncover adipose LNs as an interface for the neuro-immune crosstalk to maintain energy homeostasis, and provide a molecular explanation for the intra- and inter-depot variations in beiging capacity of WAT.

### Sympathetic innervation of LNs in cold-induced beiging of WAT

Previous cellular and anatomical studies in both humans and rodents have observed the presence and distribution of sympathetic nerves, expression of neurotransmitters and neuropeptide receptors in various LNs^37,45^. A recent publication demonstrates that lymph nodes are also innervated by a unique population of sensory neurons^46^. Retrograde tracing studies on innervation of LNs demonstrated that sympathetic input from postganglionic neurons that are associated with supplying sympathetic input to the particular region of the body where the immune organ is located^47^. The SNS and its main neurotransmitter, NE, have been proposed as the primary pathways responsible for the neural regulation of immune functions^48^. Aging and inflammation alter the pattern of lymphoid innervation, and social stress also enhances the density of sympathetic nerve fibers in axillary LNs of primates^49,50^. However, the physiological role of sympathetic innervation of LNs remains elusive. The present study shows that cold exposure increases the sympathetic innervation of inguinal LNs, whereas cold acclimatization-induced beiging of WAT is largely abrogated by surgical removal or pharmacological denervation of iLNs, as well as knockdown of β1- and β2-AR in FRCs in iLNs, suggesting that sympathetic innervation of LNs and β-AR signaling in FRCs are key physiological mediators through which the brain controls energy expenditure and thermogenesis in WAT during adaptation to environmental cold. Given that LNs are the primary immune checkpoints to identify and fight pathogen infection^51^, the thermosensitive nature of LNs with sympathetic input may represent an important mechanism regulating the function of immune cells within LNs in response to thermal/cold stress. In a cold environment, beiging and thermogenesis of WAT elicited by the SNS-LNs axis may act through a positive feedforward loop to boost the activities of immune cells within LNs to combat pathogen invasion by raising the local temperature.

### LN FRCs as a major source of IL-33 production in response to NE

IL-33 is a cytokine of the IL-1 family that is classically associated with infection and inflammatory diseases^52,53^. Recent data from both animals and humans suggest that this type 2 cytokine plays a key role in energy balance and AT homeostasis, and its dysfunction contributes to obesity and its related metabolic complications^54^. Mice with genetic ablation of IL-33 or its receptor ST2 exhibit defective beige adipocyte formation and are more susceptible to develop obesity than their wild-type littermates^11,55,56^, whilst administration of recombinant IL-33 is sufficient to reduce body weight and adipose mass in both lean and obese mice by increasing energy expenditure^11,12^. Although the critical role of IL-33 in inducing beige biogenesis and type 2 immune responses in WAT has been reported by several independent studies^11,12,35^, the cellular source of IL-33 remains elusive. A previous study has identified mesothelial cells as a possible source of IL-33 for activation of ILC2 and recruitment of eosinophils in visceral WAT upon HFD feeding^57^. However, this study did not explore whether mesothelial cell-derived IL-33 is a source of cold-induced elevation of IL-33 and is functionally relevant to beiging of WAT, which warrants further investigation. In the present study, we demonstrate that FRCs in LNs embedded within WAT, but not WAT itself is the major cellular source of IL-33 that mediates chronic cold-induced beige biogenesis and thermogenesis. This notion is supported by our findings that cold-induced elevation of IL-33 in inguinal WAT is markedly diminished by surgical removal or pharmacological denervation of iLNs, as well as genetic ablation of IL-33 or β1- and β2-AR in FRCs in iLNs, whereas replenishment of IL-33 in iLN-deficient mice largely restores cold-induced beiging of WAT. In line with our findings, a previous study using IL-33-LacZ gene trap reporter mice observed a high level of constitutive expression of IL-33 in FRCs that distribute in LNs^32^. Furthermore, a recent study has identified FRCs as a key source of IL-33-driving acute and chronic antiviral T-cell responses^33^. Taken together, these findings support the role of FRCs as sensor cells of sympathetic signals coordinating neurological and immunological responses to infection and metabolic stresses. Nevertheless, the detailed underlying mechanism whereby IL-33 derived from FRCs in iLN is being delivered to iLN-surrounding scWAT in response to cold temperature remains poorly defined. Further studies are warranted to explore the characteristics and distribution of lymphatic vessels in scWAT under different environmental or nutritional conditions.

Like other members of the IL-1 family, IL-33 lacks a classical signal peptide necessary for secretion, and it is released into the extracellular space after cellular injury, as an alarm signal to alert immune cells of tissue injury during trauma or infection^58^. Additionally, biomechanical stretching has also been reported to induce IL-33 secretion from living cells without any cell damage or death^59^. Our in vitro results reveal that the β-AR agonist isoproterenol stimulates the release of IL-33 from FRCs through the activation of the β-ARs/PKA signaling cascade. In mice, the selective knockdown of β1- and β2-AR in FRCs of iLNs leads to obvious impairments in cold-induced release of IL-33 from FRCs into scWAT, suggesting an indispensable role of the β-AR signaling in controlling IL-33 secretion in FRCs within iLNs. Similarly, the adrenoreceptor pathway has been shown to promote the secretion of IL-33 from murine bone marrow-derived dendritic cells in response to norepinephrine combined with lipopolysaccharide^60^. Stimulation of β-ARs has also been implicated in the contractility of heart muscle^61,62^ and actomyosin mechanics in brown adipocytes^63^. Taken together, these findings support the notion that cold-induced release of IL-33 from FRCs in iLNs is attributed to the activation of β1- and β2-AR signaling in FRCs probably by enhancing actomyosin contractility.

### FRC-derived IL-33 activates adipose-resident ILC2s to promote beiging of WAT

Several previous studies have shown that the stimulatory effect of IL-33 on beiging of WAT is dependent on ILC2^11,12^. IL-33-activated ILC2 triggers the conversion of preadipocytes to beige adipocytes by the production of IL-13 and IL-4 through IL-4Rα receptor and also induces UCP1 expression and oxygen consumption through secretion of MetEnk^11,12^. In line with these observations, cold-induced activation of ILC2 and production of type 2 cytokines and MetEnk in WAT is abrogated in iLNs-depleted mice, mice with FRC-specific ablation of IL-33, knockdown of β1- and β2-AR or with selective denervation of iLNs, whereas these impairments are reversed by replenishment of recombinant IL-33, suggesting an obligatory role of LNs FRC-derived IL-33 in the activation of adipose-resident ILC2 and ensuing biogenesis of beige adipocytes in WAT. In healthy and lean subjects, WAT is infiltrated with several types of type 2 immune cells (ILC2s, eosinophils and M2 macrophages) that are important for the maintenance of an anti-inflammatory environment and local metabolic homeostasis. Therefore, LNs embedded within AT may contribute to the maintenance of adipose homeostasis and metabolic health through IL-33/ILC2 axis. In line with this notion, lymphatic defects caused by Prospero-related homeobox (Prox1) haploinsufficiency leads to the adult-onset of obesity^64^, whereas restoration of lymphatic function is sufficient to reverse the obese phenotype in Prox1^+/−^ adult mice^65^. Additionally, a single nucleotide polymorphism (SNP) in the Prox1 gene is associated with obesity and type 2 diabetes in humans^66,67^, suggesting that lymphatic dysfunction may contribute to obesity and its metabolic complications. Another notable finding in our study is that cold-induced elevation of IL-33 in scWAT is only sufficient to increase ILC2 activity, whereas direct injection of rmIL-33 into scWAT at a pharmacological dose increases both the number and activity of ILC2s, suggesting a dose-dependent effect of IL-33 on ILC2 in scWAT. Increased proliferation and/or recruitment of ILC2 in scWAT occur only when IL-33 reaches to a super-physiological concentration, as reported in previous studies^11,12^.

A high degree of inter- and intra- depot variations in beiging of WAT represents a major challenge in developing anti-obese therapies by promoting biogenesis/thermogenesis of beige adipocytes. Our present study suggests such variations in the beiging capacity are attributed at least in part to the presence and distribution of LNs embedded in WAT. Indeed, the anatomical location of LNs largely overlaps with the regions of WAT that are susceptible to beiging in both humans and mice^19,68^. The presence of iLN in scWAT, but not eWAT, may explain why scWAT is more susceptible to beiging than eWAT. Consistent with our findings, previous 3D imaging studies also showed that UCP1^+^ beige adipocytes are mainly localized in the middle part of scWAT close to iLN after 48 h cold exposure and then extend from the iLN-surrounding region to distal regions after prolonged cold acclimation^14,15^. Another study has also identified a new subset population of UCP1^+^ beige adipocytes adjacent to iLN after 2 weeks of cold exposure, which are not observed after treatment of the β3-AR agonist (CL316,243)^69^. Therefore, pharmacological interventions mimicking the functions of LNs to create an immune microenvironment favorable for beige biogenesis may be a feasible therapeutic option for obesity.

In summary, our study identified FRCs in iLNs as a major source of IL-33 mediating cold-induced ILC2 activation in WAT, and uncovered an unidentified role of LNs as a key player of the neuro-immune circuits in promoting beiging and adaptive thermogenesis of WAT, beyond its classical role in protection against pathogen infection. Further elucidation of the molecular basis underlying LNs-mediated inter-organ crosstalk between brain and adipose tissues may shed new light on the intricate relationship among metabolism, immunity and infection.

## Methods

### Animal studies

All C57BL/6N mice (obtained from the Laboratory Animal Unit at the University of Hong Kong) and IL-33^fl/fl-^eGFP mice (imported from The Jackson Laboratory, Strain #030619) were housed in a controlled facility (22 ± 1 °C, 12-hour light/dark cycle, 60-70% humidity) with ad libitum access to water and either a standard chow diet (LabDiet 5053, LabDiet) or high-fat diet (45 kcal% fat, D12451, Research Diet, USA). For cold challenge experiments, 8-week-old mice were housed at thermoneutrality (30 °C) for 3 weeks in an intensive care unit (ThermoCare, California, USA) prior to exposure at 6 °C for 2 days in pre-chilled cages in a temperature and light-controlled (12 h light/dark cycle) enclosure (CLAMS-ENC52, Columbus Instruments, Ohio, USA). Animals were euthanized by cardiac puncture after anaesthetization at the end of the experiments. All animal experiments were approved by the Committee on the Use of Live Animals in Teaching and Research (CULATR, #4375-17, #5184-19) at the University of Hong Kong. The inguinal region of scWAT was used for all the analyses unless otherwise stated.

### Inguinal lymphadenectomy

Eight-week-old male C57BL/6 N mice were subjected to surgical removal of iLNs from unilateral or bilateral sides of scWAT^70^. Briefly, hair was removed from the hind paw near the abdomen after anesthetizing the mice. A 5 mm incision was made adjacent to the hind paw and stretched with 2 forceps to locate the iLN which appeared darker than the surrounding fat. iLN was removed with sterile scissors and then the surgical wound was secured using 5-0 nylon simple interrupted sutures. For the sham-operated group, only 5 mm was excised on the skin without disturbing the iLN. All the procedures were conducted in a sterile condition. After the operation, mice were placed in a warm clean cage at 30 °C for 3 weeks of post-operative recovery before further experiments.

### Generation of lentiviruses

The Ccl19 promoter sequence (3 kb upstream of the transcription start site) was chemically synthesized and then inserted into the pSLenti-CMV-MCS-WPRE vector to replace the CMV promoter, resulting in the generation of pSLenti-Ccl19-MCS-WPRE vector. Sequences of FLAG-tagged Cre recombinase and luciferase, shRNA against β1-AR or β2-AR respectively (Supplementary Data 1), or luciferase together with or without scrambled shRNA (Supplementary Data 1), were inserted into the pSLenti-Ccl19-MCS-WPRE vector to construct Lenti-Ccl19-Cre, Lenti-Ccl19-shβ1AR, Lenti-Ccl19-shβ2AR, Lenti-Ccl19-Luci and Lenti-Ccl19-scrambled vectors. These vectors were subsequently subjected to lentiviral packaging and titration to generate the corresponding lentiviruses (OBiO Technology Corp., Ltd, Shanghai).

### Injection of lentivirus into iLN

A surgical operation was first performed to enable the injection of lentivirus directly into iLN of the mice. In brief, hair was removed, and a 5 mm incision was made to locate the position of iLN. To generate iLN FRC-specific IL-33 KO mice, Lenti-Ccl19-Cre was injected into iLN of eight-week-old male IL-33^fl/fl^ mice using a microsyringe (Hamilton™) at a dosage of 7.5 × 10^6^ TU. Lenti-Ccl19-Luci was used as the control group. To generate mice with knockdown of β1-AR and β2-AR in FRCs in iLN, Lenti-Ccl19-shβ1AR and Lenti-Ccl19-shβ2AR were co-injected into iLNs of eight-week-old male C57BL/6 N mice at a dosage of 5 × 10^6^ TU for each virus. Lenti-Ccl19-scrambled was used as the control group. After injection, mice were housed at 30 °C for 3 weeks followed by 2-day cold exposure (6 °C) or continued to be housed at 30 °C for another 2 days. Mice were then sacrificed, and tissues were collected for further analyses. IVIS in vivo imaging system (Perkin Elmer, Xenogen IVIS 124262) was used to detect the localization and intensity of luciferase expression in mice after the lentivirus injection for one week by intraperitoneal injection of luciferin (150 mg/kg) for 10 min.

### Generation of LN-depleted progeny mice

Timed pregnancy in each female C57BL/6 N mouse was set up by checking the presence of vaginal plug after overnight mating. When the plug was found, it was considered as embryonic day 1 (E1). In parallel, body weight was also recorded every 2 days to confirm pregnancy. Timed pregnant female mice were then intravenously (i.v.) injected with 200 μg of LTβR-IgG2α fusion protein (Genetech) or control mouse IgG2α isotype (Invitrogen) on E13 and E14 as described^42^ with minor modifications.

### In vivo study with replenishment of rmIL-33 protein

Eight-week-old male C57BL/6 N mice after unilateral or bilateral iLNX or sham operation were housed at 30 °C for three weeks. 250 ng endotoxin-free recombinant mouse IL-33 protein (rmIL-33) (Immunodiagnostics, Hong Kong) dissolved in 100 ul phosphate-buffered saline (PBS) was subcutaneously injected into scWAT for 4 consecutive days. Mice were then subjected to cold exposure (6 °C) or continued to be housed at 30 °C for 2 days after the first two injections and were sacrificed on day 4 to collect tissues for further analyses.

### Sympathetic denervation of iLN

Surgical operation was performed as mentioned above to unilaterally or bilaterally inject 6-OHDA directly into iLN of the C57BL/6 N mice. 3 μl of 6-OHDA (9 mg/ml in 0.15 M NaCl) was injected into iLN using a microsyringe (Hamilton™) and the surgical wounds were secured. After 3 weeks of post-operative recovery at thermoneutrality (30 °C), mice were subjected to cold challenge (6 °C) or continuously housed at 30 °C for another two days. Mice were then sacrificed, and tissues were collected for further analyses.

### Intranodal injection of fluorescent tracer

Eight-week-old male C57BL/6 N mice were anesthetized followed by the surgical operation as mentioned above to expose iLN to the injection of soluble fluorescein-conjugated dextran (40 kDa) (Molecular Probes/Invitrogen, #D1845). 2 μl of PBS containing 1.25 μg soluble fluorescein-conjugated dextran was directly injected into iLN using microsyringe (Hamilton™). iLNs were harvested after injection for 2 or 20 min, respectively, followed by embedding in Tissue-Tek® O.C.T. compound, snapped frozen and stored at −80 °C until ready for cryostat sectioning.

### Ex vivo oxygen consumption assay

Oxygen consumption assay in adipose tissues was performed using a Seahorse XFe24 extracellular flux analyzer (Agilent Technologies) as described previously^8^. Briefly, freshly excised scWAT and BAT were weighed and cut into 2 mm^3^ pieces before placing in each well of a Seahorse XF24 Islet Capture plate. Prior to the assay, minced tissues were equilibrated in Seahorse XF base medium supplemented with 2% BSA, 25 mM glucose, 1.5 mM sodium pyruvate and glutamine, with pH adjusted to 7.4 at 37 °C for an hour. Basal oxygen consumption rate (OCR) was measured and normalized with tissue weight.

### Flow cytometry

The stromal vascular fraction (SVF) was isolated as previously described^9^. In brief, scWAT was minced into pieces with scissors before digesting in 2 mg/ml of Collagenase I buffer (DMEM supplemented with 3% of BSA) for 30 min at 37 °C. Digested scWAT were filtered through a 70 μm cell strainer (BD Biosciences) and centrifuged at 800 g for 10 min at 4 °C to separate SVF from adipocyte fraction. The SVF pellets were incubated with red blood cell ammonium-chloride-potassium (ACK) lysis buffer for 1 min on ice, followed by centrifugation at 800 g for 10 min at 4 °C and washed with PBS. Single cell suspension was stained with live/dead fixable dead cell stain (Molecular Probes) in PBS for 30 min on ice. Subsequently, cells were washed with FACS buffer (1% BSA in PBS) and preincubated with 3% BSA for another 30 min on ice before immunostaining with appropriate antibodies. For the analysis of ILC2s, suspended cells were stained with FITC lineage cocktail (anti-Cd3e, -Ly-6G, -Ly-6C, -Cd11b, -Cd45R/B220, -Ter-119) (1:200, clone 145-2C11, RB6-8C5, RA3-6B2, Ter-119, M1/70, Biolegend #133301), rat monoclonal antibodies (mAbs) against mouse: Cd5-FITC (1:100, clone 53-7.3, Biolegend #100605), Cd45-PerCP-Cyanine5.5 (1:100, clone 30-F11, eBioscience #45-0451-82), IL33R- Phycoerythrin (PE) (1:100, clone U29-93, BD Biosciences #566311) and Cd127-PE-Cy^TM^7 (1:100, clone SB/199, BD Biosciences #560733). For the identification of eosinophils, cells were incubated with rat mAbs against mouse: Cd45-PerCP-Cyanine5.5, F4/80-PE (1:100, clone CI:A3-1, Abcam #ab105156), Cd11b-BV421 (1:100, clone M1/70, BD Biosciences #562605) and Siglec F-Alexa Fluor® 647 (1:100, clone E50-2440, BD Biosciences #562680). For analyses of macrophages, suspended cells were stained with rat mAbs against mouse: F4/80-FITC (1:100, clone BM8, Abcam #ab60343), Cd11b-BV421 (1:100), Cd206-Alexa Fluor® 647 (1:100, clone C068C2, Biolegend #141712) and Armenian Hamster mAb against mouse Cd11c-PE (1:100, clone N418, Biolegend #117308). For the analysis of γδ T cells, suspended cells were incubated with rat mAbs against mouse: Ter119-FITC (1:100, clone Ter119, Biolegend #116205), F4/80-FITC, Cd19-FITC (1:100, clone 1D3/CD19, Biolegend #152403), Cd45-Pacific Blue, and Armenian Hamster mAbs against mouse: TCRγ/δ-APC (1:50, clone eBioGL3 (GL-3, GL3), eBioscience # 17-5711-81) and Cd3e-PE (1:50, clone145-2C11, eBioscience #12-0031-81). For the analysis of neutrophils, cells were stained with rat mAbs against mouse: F4/80-FITC, Cd11b-BV421, Ly6G-BV711 (1:100, clone 1A8, Biolegend #127643) and Armenian Hamster anti-mouse Cd11c-PE/Cyanine7 (1:100, clone N418, Biolegend #117318). For the identification of dendritic cells, suspended cells were incubated with mouse anti-mouse Cd64-APC (1:50, clone X54-5/7.1, Biolegend #139305), Cd45-PerCP-Cyanine5.5, and Cd11c-PE. For the analysis of adipocyte precursors, cells were incubated with FITC lineage cocktail (1:200), rat mAbs against mouse: Cd45-FITC (1:100, clone 30-F11, Biolegend #103107), Sca1-Pacific blue^TM^ (1:100, clone D7, Biolegend #108120) and CD81-APC (1:100, clone Eat-2, Biolegend #104909). All the antibodies were incubated in the dark for 30 min on ice. For the analysis of beige adipocyte precursors, cells were incubated with FITC lineage cocktail, rat mAbs against mouse: Cd45-FITC, Cd140α (Pdgfrα)-BB700 (1:100, clone APA5, BD Biosciences #742176), and Sca1-Pacific blue^TM^ in the dark for 30 min on ice. Cells were then fixed and permeabilized using the Fixation/Permeabilization kit (eBioscience #00-5523) according to the manufacturer’s instructions before staining with rat mAb against mouse Cd137-PE (1:100, clone 1AH2, BD Biosciences #558976) and rabbit anti-mouse Tmem26 (1:200, Novus Biologicals #NBP2-27334SS) followed by staining with a secondary PE-Cy7 goat anti-rabbit antibody (1:100, Santa Cruz Biotechnology #sc-3845) on ice for 30 min in dark. All samples were fixed with 2% paraformaldehyde (PFA) and stored at 4 °C in PBS before analysis. For the analysis of stromal cells in LN, cells were isolated from IL-33^fl/fl^-eGFP mice and were then triple-stained with rat mAb anti-Cd45-Pacific blue (1:100, clone 30-F11 Biolegend #103126), rat mAb anti-Cd31-PE-Cy7 (1:50, clone 390, Biolegend #102418) and Syrian hamster mAb anti-podoplanin (gp38)-APC (1:100, clone 8.1.1 Biolegend #127410). Subsequently, cells were fixed and permeabilized before staining with rabbit mAb anti-Cre recombinase (1:400, Cell Signaling Technology, D3U7F) followed by a secondary goat anti-rabbit Alexa Fluor® 568 antibody (1:800, Thermo Fisher Scientific, #A11011). All samples were subjected to BD LSRFortessa Cell Analyzer (BD Biosciences). Data were analyzed using FlowJo software version X.0.7 (Tree Star, Inc.). Gates were drawn according to respective fluorescence minus one (FMO) controls.

### Intracellular staining for cytokines and MetEnk

The production of intracellular cytokines from ILC2s was detected as previously described^11^. Isolated scWAT SVF, as described above, were cultured in RPMI-1640 media supplemented 10% FBS containing phorbol 12-myristate 12-acetate (PMA, 100 ng/ml), ionomycin (1 μg/ml) and brefeldin A (10 μg/ml) in an incubator (5% CO_2_, 37 °C) for 4 h. Cells were then centrifuged, resuspended and stained with cell viability dye followed by surface markers staining for ILC2s (Lin^-^Cd5^-^Cd45^+^Cd127^+^IL33R^+^). Subsequently, cells were fixed and permeabilized before double-staining with rat mAbs against mouse: IL-5 APC (1:100, clone TRFK5, BD Biosciences #554396), and IL-13 PE-eFluor® 610 (1:100, clone eBio13A, eBioscience #61-7133-82) intracellular cytokines. For the staining of MetEnk, single cell suspensions were also stimulated with PMA (100 ng/ml) and ionomycin (1 μg/ml) in the presence of brefeldin A (10 μg/ml) and monensin (2 μM). Cells were stained for rabbit pAb against mouse MetEnk (1:300, Bioss Inc. #bs-1759R) followed by staining with a secondary BV421 donkey anti-rabbit antibody (1:100, clone Poly4064, Biolegend #406410).

### Measurement of NE content

scWAT (~50 mg wet weight) and iLNs (~1 mg wet weight for each) were weighed and homogenized in 200 μl perchloric acid (0.3 M) containing 0.3 mg/ml ascorbic acid and 2 μl of internal standard (NE-D6, 1 ng/μl) for 5 min (with 1 min interval on ice after 1 min homogenization). After centrifugation at 13,000 g for 10 min at 4 °C, the supernatant was filtered through a 0.2 μm syringe filter into a sample tube before injecting into an Agilent 6460 Triple Quadrupole LC/MS system (Agilent Technologies). The collision energy and collision cell accelerator were set to 15 V and 7 V, respectively. The separation of NE was carried out on an Amide column (particle size, 1.7 μm; 2.1 mm × 100 mm) purchased from Waters™. NE content was calculated according to the linear NE standard curve (standard concentrations: 10 nM, 25 nM, 50 nM, 250 nM, 1 μM, 5 μM) and normalized with tissue weight.

### Measurement of NE turnover (NETO)

Eight-week-old male C57BL/6 N mice were housed at 30 °C for three weeks prior to either thermoneutral (30 °C) or cold (6 °C) condition for 48 h. Mice were intraperitoneally injected with alpha methyl-para-tyrosine (αMPT, 300 mg/kg body weight), a competitive inhibitor of tyrosine hydroxylase (TH). A supplemental dose of αMPT (150 mg/kg body weight) was administered to these mice 2 h after the initial injection to sustain the inhibition of TH. For baseline NETO, vehicle control groups were injected with saline. The mice were then sacrificed 4 h after the initial α-MPT injection. iLN, scWAT close to iLN and BAT were harvested, weighed and subjected to NE content measurement using LC/MS system and calculation was based on the following equations: *k* = (log[NE]_0_-log[NE]_4_) / (0.434*4) and NETO = k*NE_0_^71^. NE_0_ is the NE content of the saline-injected groups and NE_4_ is the NE content of the αMPT-injected groups. The NETO was obtained by multiplying the rate of NE efflux (k) by the initial NE content of baseline animals^72,73^.

### Whole-mount immunofluorescence staining

iLNs were fixed in 4% PFA for 48 h after isolation from mice that underwent myocardial perfusion with PBS followed by 4% PFA. Afterwards, iLNs were immersed in 8% SDS for at least 2 days at 37 °C, to ensure the whole iLN become transparent. iLNs were then washed with PBS containing 0.1% Tween 20 (PBST) for 3 times, followed by incubation with primary antibodies: rabbit pAb against mouse TH (1:200, Merck Millipore #ab152) and rat mAb against mouse Cd3 (1:200, clone CD3-12, Abcam #ab11089) for 2 days. Afterwards, iLNs were washed with PBST for 5 times and incubated with goat anti-rabbit Alexa Fluor® 488 (1:400, Thermo Fisher Scientific, #A11008) and goat anti-rat Alexa Fluor® 568 antibodies (1:400, Thermo Fisher Scientific, #A11077) for another 2 days, followed by washing with PBST and incubation with 88% histodenz (Sigma-Aldrich) for at least 24 h prior to confocal microscopy analysis with tile scanning and Z-stack using Carl Zeiss LSM 780. The captured images were merged and analyzed using ZEN software.

### Immunofluorescence and immunohistochemical staining

Adipose tissues were fixed with 4% PFA solution for 24 h, dehydrated, and then embedded in paraffin wax using Paraffin embedding station EG1150 (Leica Biosytems). Deparaffinized and dehydrated sections (~5 μm thickness) were stained with hematoxylin-eosin, or rabbit pAb against mouse UCP1 (1:500, Abcam, ab10983) as previously described^8^. Images were captured using an Olympus biological microscope BX41 with DP72 color digital camera. For immunofluorescence staining, cells or tissues were fixed with 4% PFA for 20 min followed by incubation with the following primary antibodies: rabbit pAb anti-Cre (1:200, Novagen #69050), mouse mAb anti-FLAG (1:200, Sigma-Aldrich #F3165), rabbit mAb anti-perilipin (1:200, Cell Signaling Technology #9349 S), rabbit pAb anti-β1-AR (1:200, clone V-19, Santa Cruz Biotechnology #C1313), rabbit mAb anti-β2-AR (1:200, clone EPR707(N), Abcam #ab182136), goat pAb anti-IL-33 (1:200, R&D Systems #AF3626), F-actin fluorescent probe SPY555-actin (1:1000, Spirochrome #CY-SC202), Syrian hamster mAb anti-gp38 (1:200, eBio8.1.1 (8.1.1), eBioscience #14-5381-82), rabbit pAb anti-Lyve1 (1:100, Abcam, ab14917) or rabbit mAb anti-Cd31 for overnight at 4 °C. Sections were then incubated with respective fluorochrome-conjugated secondary antibodies diluted in 3% BSA for an hour at room temperature. The slides were counterstained with Hoechst 33342 Fluorescent Stain (0.1 μg/ml), mounted with an aqueous mounting medium (Dako Faramount) and photographed under Carl Zeiss LSM710 or LSM780 laser scanning confocal microscope. Gap analysis of FRCs was performed using a MATLAB script as described previously^38^. The distribution of circle radius was plotted. To quantify the distribution of F-actin in FRCs, region of interest (ROI) was created by drawing a straight line perpendicular with the long axis of a cell, capturing the cell edge, cytoplasm and nucleus using Zeiss ZEN software (version Blue 3.1). This produced a fluorescence intensity (FI) histogram vs distance (μm) for the red (F-actin), green (IL-33) and blue (Nucleus) acquisition channels along the straight-line ROI. Average FI of cell edge (<5 μm) and cytoplasm (>5 μm) were calculated.

### Isolation, culture and pharmacological treatment of FRCs

FRC cell lines were established from peripheral LNs by long-term culturing as described previously^74^ with minor modifications. In brief, LNs from 8-week-old C57BL/6 N mice were dissected and disrupted using two 25 G needles before enzymatic digestion with DMEM medium containing 3.5 mg/ml Collagenase D and 40 μg/ml DNase I at 37 °C for 30 min with agitation^75^. The mixture was then filtered through a 70 μm cell strainer and centrifuged at 300 g for 5 min at 4 °C. The cell pellet was resuspended and cultured in DMEM medium (supplemented with 10% FBS and 1% Penicillin/Streptomycin) (5% CO_2_, 37 °C). After 24 h, non-adherent cells were removed, and fresh medium was added to continue culturing until cells reached confluence. Adherent-stromal cells were then trypsinized, and triple-stained with antibodies to identify FRCs: Cd45 Pacific blue (1:100), Cd31 PE (1:100) and gp38 APC (1:100). FRCs were sort-purified using a MoFlo Optical Bench Sorter (Beckman Coulter) to achieve a purity of ≥95%. The sorted cells were immediately cultured in DMEM medium for expansion, and then seeded into 60 mm dishes in a density of 3 × 10^5^ per well to grow until confluence, followed by starvation for overnight and treatment with 10 μM isoproterenol (Sigma-Aldrich), 5 μM forskolin (Sigma-Aldrich), and 5 μM PKA inhibitor H89 dihydrochloride hydrate (Sigma-Aldrich) for 8 h. Culture media were collected to determine the concentration of IL-33 using a mouse IL-33 immunoassay kit (Immunodiagnostics Limited) or LDH using a CyQUANT LDH Cytotoxicity fluorescent assay kit (Thermo Fisher Scientific).

### Measurement of rectal and surface temperatures

Core body temperature of mice was measured by a thermometer with rectal probe (Model 4610 Precision Thermometer, Measurement Specialties) at 0, 2, 4, 8, 24 and 48 h. Body surface temperatures at scWAT and interscapular regions were measured without anesthesia using a FLIR T440 infrared camera. FLIR Tools® analysis software was used to quantify the surface temperatures.

### Western blot and ELISA

Proteins were extracted from adipose tissues, iLNs or FRCs using freshly prepared Radioimmunoprecipitation assay (RIPA) buffer (25 mM Tris-HCl pH 7.6, 150 mM NaCl, 5 mM EDTA, 1% NP-40, 1% sodium deoxycholate, 0.1% SDS) containing protease inhibitor cocktail (Roche). Protein mixtures were separated by SDS-PAGE, transferred onto polyvinylidene fluoride (PVDF) membranes (Bio-Rad), and then probed with primary antibodies against UCP1 (1:2500, Abcam), HSP90 (1:2500, Cell Signaling Technology, #4874 S), IL-33 (1:2500), TH (1:2500, Merck Millipore), β1-AR (1:500, Santa Cruz Biotechnology), β2-AR (1:2500, Abcam), β3-AR (1:1000, Santa Cruz Biotechnology #sc-515763) and GAPDH (1:2500, Cell Signaling Technology, #2118 L), followed by incubation with corresponding horseradish peroxidase (HRP)-conjugated secondary antibodies (Cell Signaling Technology). The protein bands were visualized with enhanced chemiluminescence reagents (GE Healthcare) and quantified using the NIH ImageJ software. The uncropped and unprocessed scans of Western blots are provided in the Source Data file. For ELISA analysis, scWAT from fresh-frozen samples were homogenized in buffer containing 1 M NaCl, 10 mM HEPES (pH7.4), and 0.5% Triton X-100 with the cocktail of protease inhibitors (Roche). Protein lysate was collected after centrifugation at 12,000 g for 10 min at 4 °C and measured using mouse IL-33 immunoassay kit (Immunodiagnostics Limited). The concentrations of IL-33 in scWAT were normalized against total protein concentrations.

### RNA preparation and real-time PCR

Total RNA was extracted from cells, frozen scWAT or iLNs using RNAiso Plus (Takara) and reverse transcribed into complementary DNA using PrimeScript RT reagent kit (Takara). Quantitative real-time PCR was performed using SYBR Premix Ex Taq (Takara) with specific primers (listed in Supplementary Data 1) on a StepOne System (Applied Biosystems). The relative gene expression level was normalized to the 36B4 gene.

### Statistical analysis

All data were statistically analyzed in GraphPad Prism 7 (GraphPad, San Diego, CA). Data were presented as mean ± standard error of the mean (SEM). All experiments were repeated at least three times with representative data shown. Statistical significance was determined using unpaired two-tailed Student’s *t*-test and Mann–Whitney *U* test. All *p* values are two-sided and *p* value of < 0.05 was considered statistically significant.

### Reporting summary

Further information on research design is available in the Nature Portfolio Reporting Summary linked to this article.

## Supplementary information

Supplementary Information

Description of Additional Supplementary Files

Supplementary Data 1

Reporting Summary

## Data Availability

All data are available within the Article, Supplementary Information and Source Data file. Source data are provided with this paper. All materials generated in the current study are available from the corresponding authors on request. Source data are provided with this paper.

## Source data

Source Data
