# Supplementary material for: Fibroblastic reticular cells in lymph node potentiate white adipose tissue beiging through neuro-immune crosstalk in male mice
Source: Nat Commun. 2023 Mar 3;14:1213. doi: 10.1038/s41467-023-36737-0 (PMC9984541; doi:10.1038/s41467-023-36737-0)
Supplement: Supplementary file 1 — Supplementary Information [file 41467_2023_36737_MOESM1_ESM.pdf]

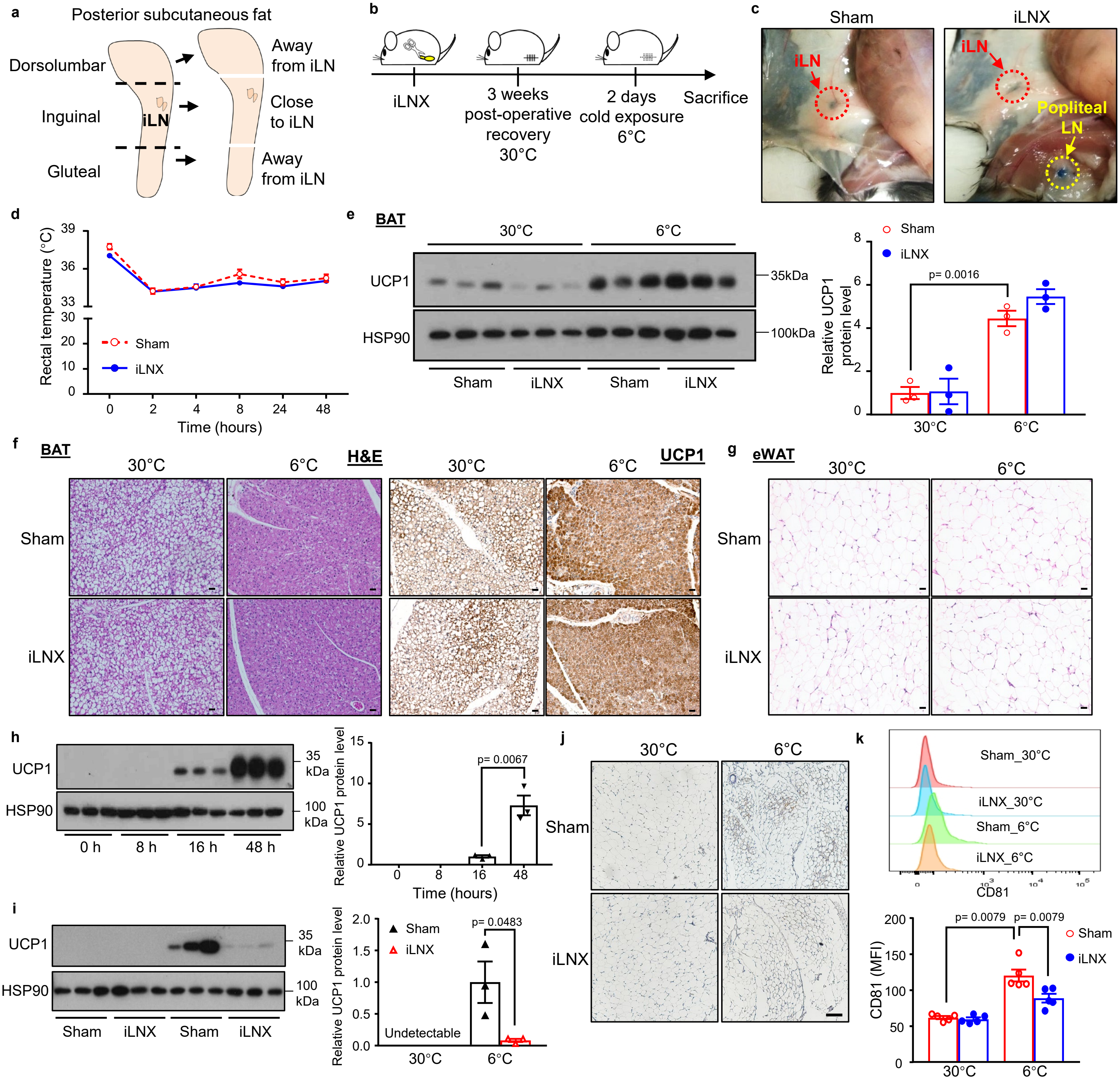

Fig. S1. Related to Fig. 1. Effects of inguinal lymphadenectomy (iLNX) on acute and chronic cold-induced beiging and thermogenesis in mice. Eight-week-old iLNX or sham-operated male C57/BL6N mice were housed at thermoneutral environment (30°C) for 3 weeks, followed by exposure to cold (6°C) or thermoneutral temperature (30°C) for 2 days. **(a)** Graphical illustration of different regions in scWAT dissected for the assessment of beiging. **(b)** Schematic diagram for the experimental protocols and timeline of this study. **(c)** Macroscopic photos of inguinal and popliteal LNs of sham-operated and iLNX mice labeled with 1% Evans blue. No blue labeling was observed at the iLN region (red arrow) after the surgery in iLNX group of mice. **(d)** Rectal temperature of mice measured at different time points during 2 days of cold exposure (6°C) (n = 5). **(e)** Western blot analysis of UCP1 protein expression in BAT of iLNX or sham-operated mice under 30°C or 6°C. The lower panel is the densitometric analysis of the relative abundance of UCP1 normalized with HSP90 (n = 3). **(f)** Representative images of H&E staining and DAB staining of UCP1 in BAT sections. Scale bar, 20 μm. **(g)** Representative images showing H&E staining of eWAT. **(h-i)** Western blot analysis of UCP1 protein level in scWAT after cold for different durations (8h, 16h and 48h) (h) or in mice subjected to bilateral inguinal lymphadenectomy (iLNX) followed by 16-hour cold exposure (i). The right panels are the densitometric analysis for the relative abundance of UCP1 normalized with HSP90 (n = 3). **(j)** Immunohistochemical (IHC) staining for UCP1 in scWAT of mice after 16-hour cold challenge. **(k)** Representative histogram overlays of flow cytometric analysis for adipocyte progenitors (APCs) ($Lin^{-}Cd45^{-}Cd31^{-}Sca1^{+}CD81^{+}$ (top) and quantification of MFI for CD81 (right) in APCs (n = 5). Scale bar, 100 μm. All samples are biologically independent replicates. Data are presented as mean ± SEM. Statistical data were assessed using unpaired two-tailed Student's t test (**e, h-i**) or Mann-Whitney U test **(k).** All the p values were two-sided. Source data are available as a Source Data file**.** kDa, relative molecular weight in kilodalton.

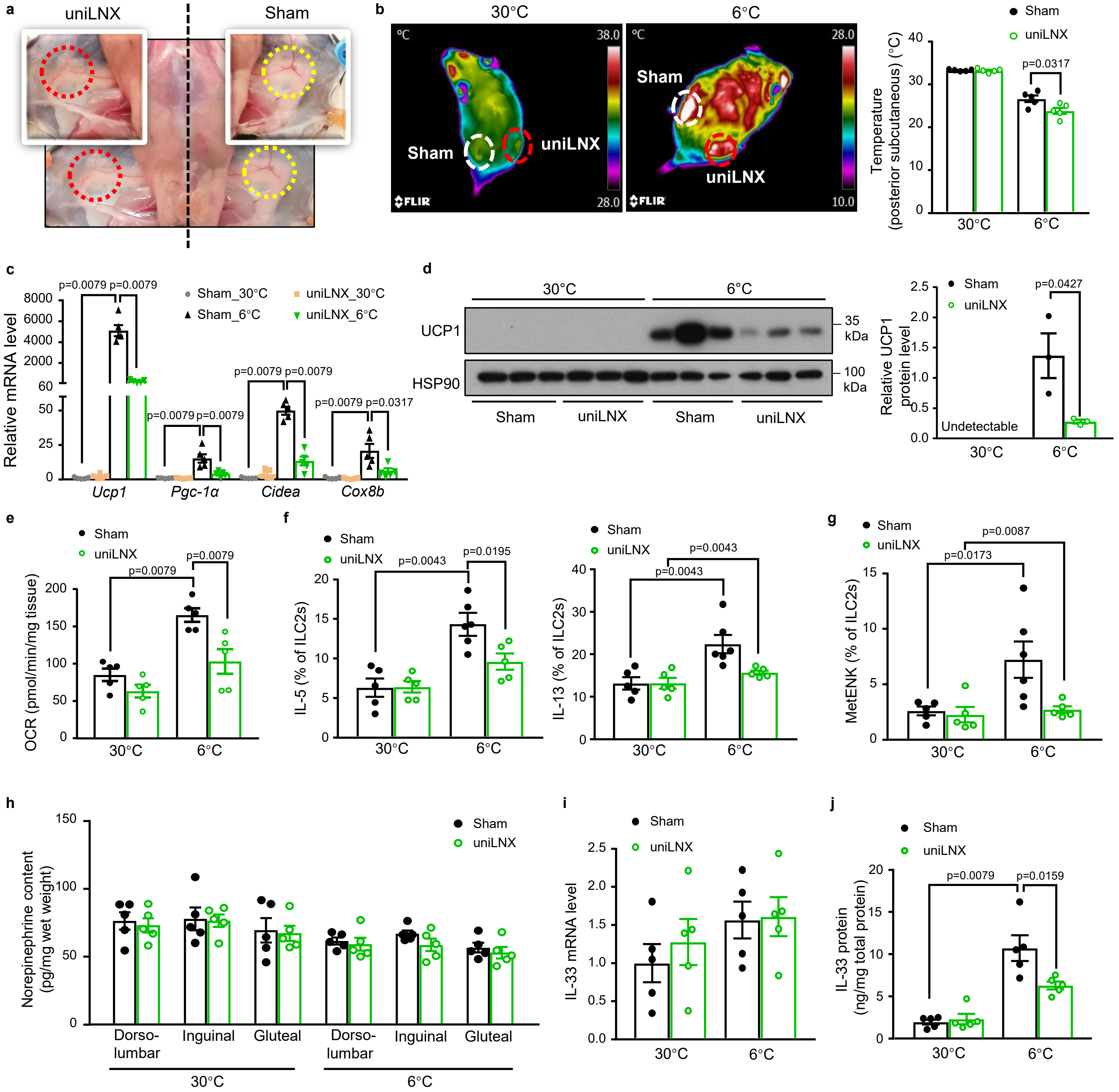

Fig. S2. Related to Fig. 1, 2, 3. Unilateral lymphadenectomy has no impact on the contralateral iLN and scWAT. Eight-week-old male C57BL/6N mice were subjected to unilateral lymphadenectomy (uniLNX) or sham operation and housed at thermoneutral environment (30°C) for 3 weeks. Then, the mice were subjected to exposure at 6°C or 30°C for 2 days. **(a)** Macroscopic appearance of scWAT with (yellow-dotted circle) or without embedded-iLN (red-dotted circle) in uniLNX mice. **(b)** Representative infrared images of uniLNX mice housed at 30°C or 6°C. Quantification of the average surface temperature in the posterior subcutaneous region (n = 5). **(c-d)** The mRNA level of several thermogenic genes (c) (n = 5) and UCP1 protein expression (d) in scWAT. The right panel in (d) is the densitometric quantification for the relative abundance of UCP1 normalized with HSP90 (n = 3). **(e)** Basal oxygen consumption rate (OCR) in the explants of scWAT (n = 5). **(f-g)** Quantification of the percentage of IL-5 and IL-13 (f), and MetENK-positive cells (g) in activated ILC2s from scWAT using flow cytometric analysis. For Sham_30°C, iLNX_30°C and iLNX_6°C (n = 5) or Sham_6°C (n = 6). **(h)** Norepinephrine content in different regions of scWAT determined by LC/MS analysis (n = 5). **(i-j)** IL-33 mRNA level (i) and ELISA analysis of IL-33 protein level (j) in scWAT (n = 5). All samples are biologically independent replicates. Data are presented as mean ± SEM. Statistical data were assessed using unpaired two-tailed Student's t test (**d**) or Mann-Whitney U test **(b-c, e-g, i-j).** All the p values were two-sided. Source data are available as a Source Data file**.** kDa, relative molecular weight in kilodalton.

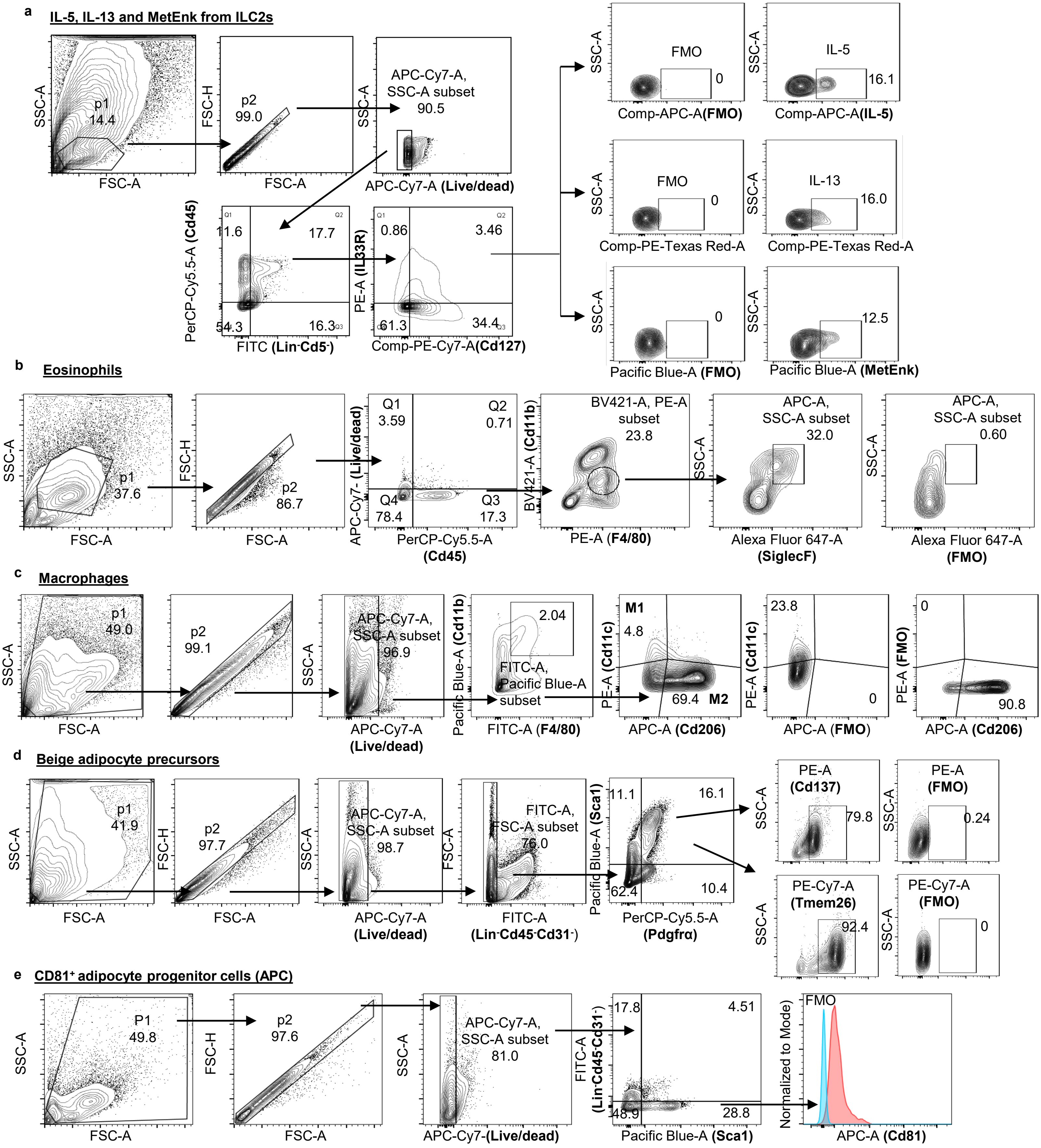

Fig. S3. Related to Fig. 2. Gating strategies for the identification of ILC2s, eosinophils, macrophages, beige adipocyte precursors and CD81$^+$ adipocyte progenitor cells in SVF from scWAT. SVF was isolated from scWAT of 11-week-old iLNX or sham-operated male C57BL/6N mice after housing at 30°C for 3 weeks and subsequently subjected to 30°C or 6°C for 2 days. **(a)** ILC2s were identified as Lin$^-$ (Cd3e$^-$Ly6G$^-$Ly6C$^-$Cd11b$^-$ Cd45R/B220$^-$Ter-119$^-$) Cd5$^-$Cd45$^+$Cd127$^+$IL33R$^+$. IL-5, IL-13 and MetEnk were further gated, respectively, based on each FMO as a gating control. **(b)** Eosinophils were identified as Cd45$^+$Cd11b$^+$F4/80$^+$SiglecF$^+$SSC^{hi} cells. **(c)** M1 macrophages were identified as F4/80$^+$Cd11b$^+$Cd11c$^+$ cells, whereas M2 macrophages were identified as F4/80$^+$Cd11b$^+$Cd206$^+$ cells. **(d)** Beige adipocyte precursors were identified as (Lin$^-$Cd45$^-$Cd31$^-$Sca1$^+$Pdgfrα$^+$Tmem26$^+$ or Cd137$^+$) cells. **(e)** CD81+ adipocyte progenitor cells were identified as (Lin$^-$Cd45$^-$Cd31$^-$Sca1$^+$CD81$^+$) cells.

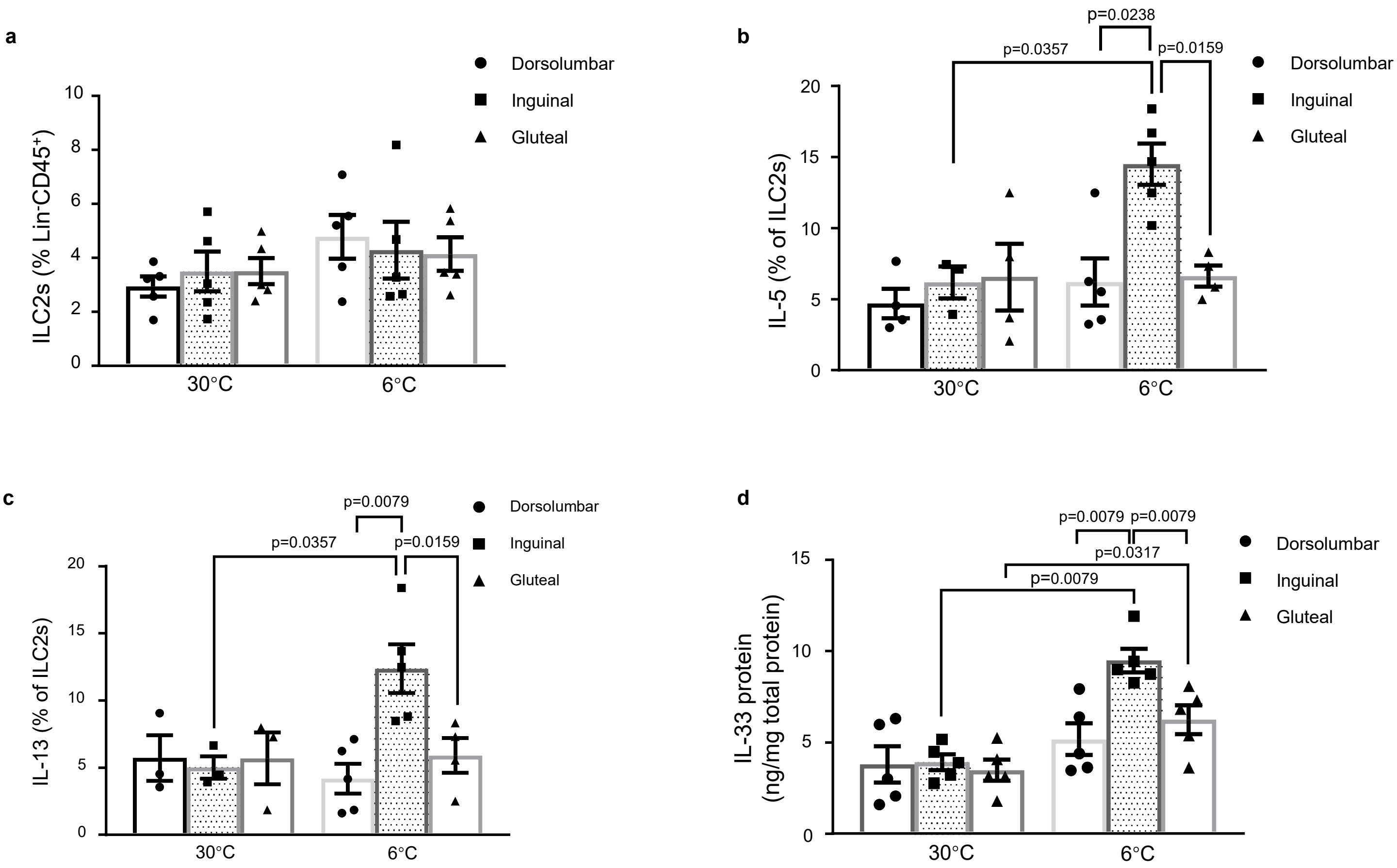

Fig. S4. Related to Fig. 2. The abundance and activation of ILC2 in different regions of scWAT. Eight-week-old male C57BL/6N mice were housed at thermoneutral environment (30°C) for 3 weeks and then subjected to exposure at 6°C or 30°C for 2 days. Different regions of scWAT (dorsolumbar, inguinal and gluteal) were collected for further analyses. **(a-c)** ILC2 number (a) (n = 5) and ILC2-derived IL-5 (b) (n = 4 for Dorsolumbar_30°C, Gluteal_30°C and Gluteal_6°C; n = 3 for Inguinal_30°C; n = 5 for Dorsolumbar_6°C and Inguinal_6°C) and IL-13 (c) (n = 3 for Dorsolumbar_30°C, Inguinal_30°C and Gluteal_30°C; n = 5 for Dorsolumbar_6°C and Inguinal_6°C; n = 4 for Gluteal_6°C) in scWAT were determined by flow cytometric analysis. **(d)** IL-33 protein level in scWAT as measured by ELISA (n = 5). All samples are biologically independent replicates. Data are presented as mean ± SEM. Statistical data were assessed using the Mann-Whitney U test **(b-d).** All the p values were two-sided. Source data are available as a Source Data file**.**

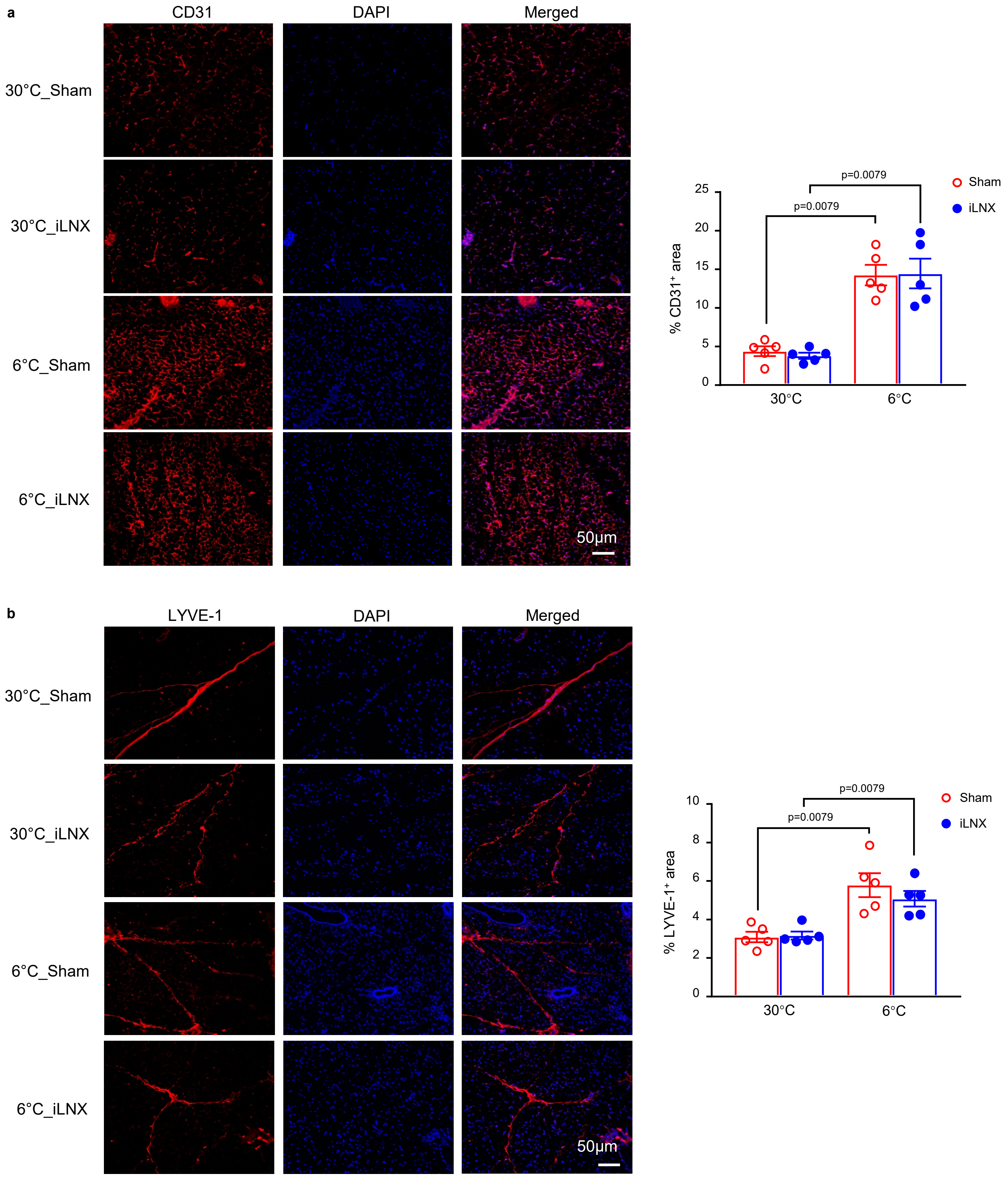

Fig. S5. Related to Fig. 2. Surgical depletion of iLN has no impact on the density of lymphatic and blood vessels in iLN-surrounding scWAT. (a-b) Representative confocal images of immunofluorescence staining for the blood vessel marker CD31 (a, red) or lymphatic vessel marker LYVE-1 (b, red) in scWAT of iLNX or sham-operated mice under 30°C or 6°C for 2 days. The right panels are the quantification of $CD31^+$ or $LYVE\text{-}1^+$ signals, respectively (n = 5 biologically independent replicates). Scale bar, 50 μm. Data are presented as mean ± SEM. Statistical data were assessed using Mann-Whitney U test **(a-b).** All the p values were two-sided. Source data are available as a Source Data file.

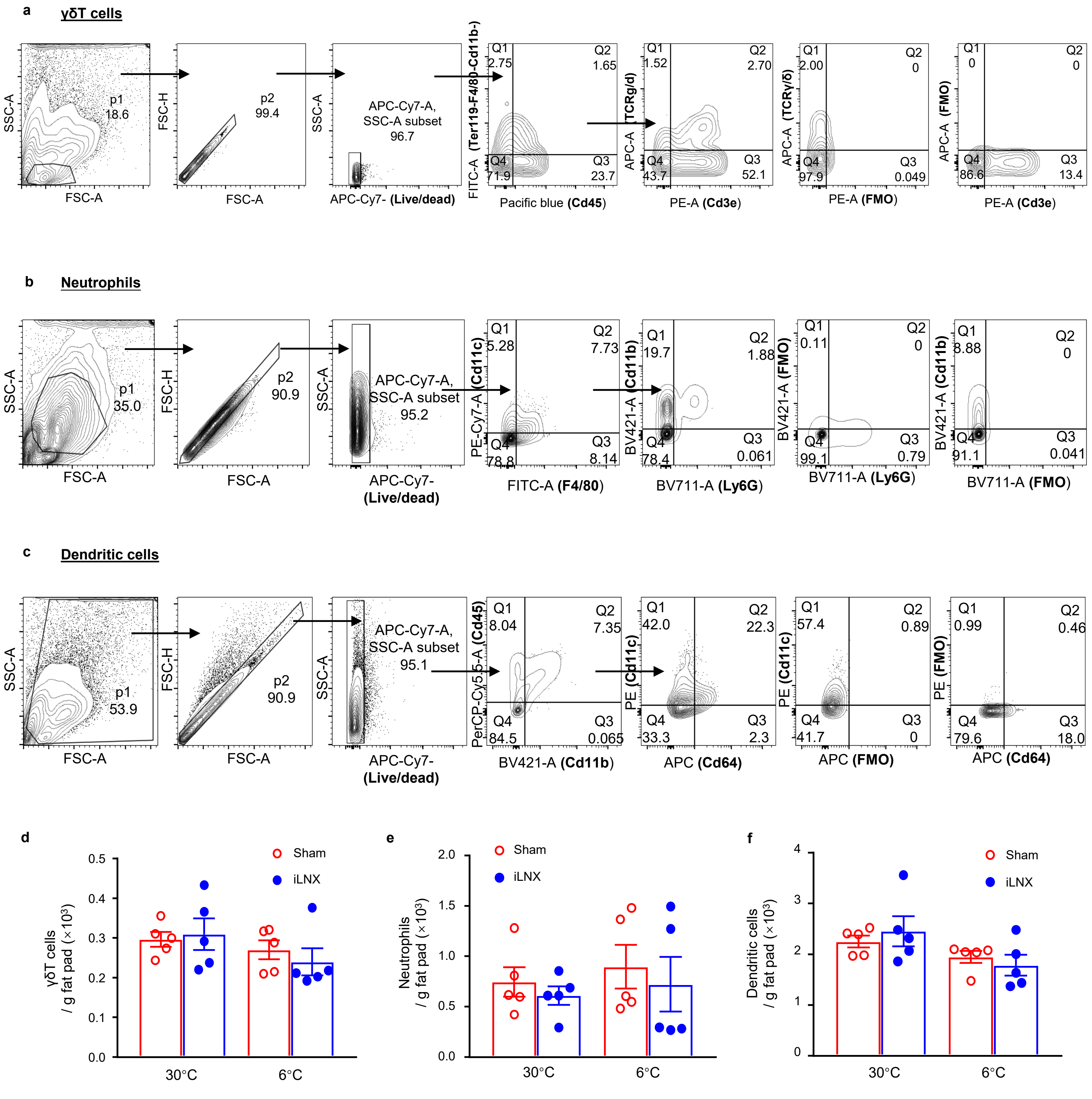

Fig. S6. Related to Fig. 2. Surgical depletion of iLN has no impact on the recruitment of γδT cells, neutrophils and dendritic cells in iLN-surrounding scWAT. (a) γδT cells were identified as Ter119$^-$F4/80$^-$Cd19$^-$Cd45$^+$TCRγ/δ$^+$CD3e$^+$ cells. **(b)** Neutrophils were identified as Cd11c$^-$F4/80$^-$Cd11b$^+$Ly6G$^+$ cells. **(c)** Dendritic cells were identified as CD45$^+$CD11b$^+$CD64$^-$CD11c$^+$ cells. **(d-f)** Absolute numbers of γδT cells (d), neutrophils (e) and dendritic cells (f) in entire scWAT as determined by flow cytometric analysis (n = 5 biologically independent replicates). Data are presented as mean ± SEM. Source data are available as a Source Data file**.**

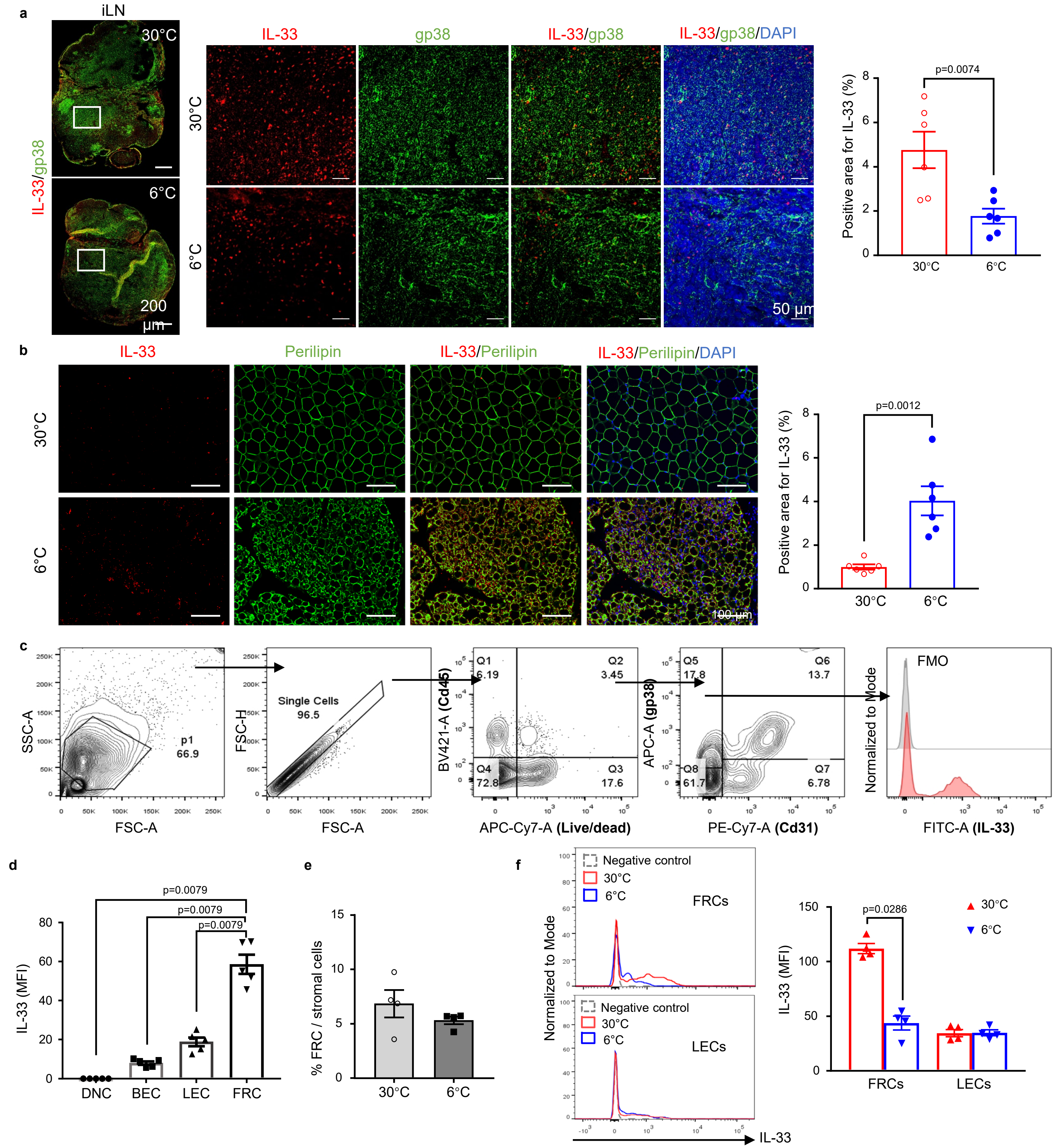

Fig. S7. Related to Fig. 3. Expression of IL-33 in scWAT and FRCs of iLN. (a-b) Representative confocal images of immunofluorescence staining for IL-33 (red) and perilipin (green) or gp38 (green) under 30°C or 6°C in iLN (a) and scWAT (b) with quantification of IL-33-positive area (right panels) (n = 6). **(c-e)** Single cells were isolated from iLN of 8-week-old male C57BL/6N mice. **(c)** Gating strategies to identify IL-33-expressing stromal cells ($Cd45^-$) in iLNs. BECs, Blood endothelial cells ($Cd45^-Cd31^+gp38^-$); LECs, Lymphatic endothelial cells ($Cd45^-Cd31^+gp38^+$); FRCs ($Cd45^-Cd31^-gp38^+$); DNCs, Double negative cells ($Cd45^-Cd31^-gp38^-$). **(d)** Quantification of Median Fluorescence Intensity (MFI) for IL-33 in DNCs, BECs, LECs and FRCs of iLN using flow cytometric analysis (n = 5). **(e)** Quantification of the number of FRCs (%) in stromal cells of iLN at 30°C or 6°C using flow cytometric analysis (n = 4). **(f)** Representative histogram overlays of flow cytometric analysis for IL-33 (left) and quantification of MFI for IL-33 (right) in FRCs and LECs, respectively (n = 4). All samples are biologically independent replicates. Data are presented as mean ± SEM. Statistical data were assessed using unpaired two-tailed Student's t test (**a-b**) or Mann-Whitney U test **(d, f).** All the p values were two-sided. Source data are available as a Source Data file.

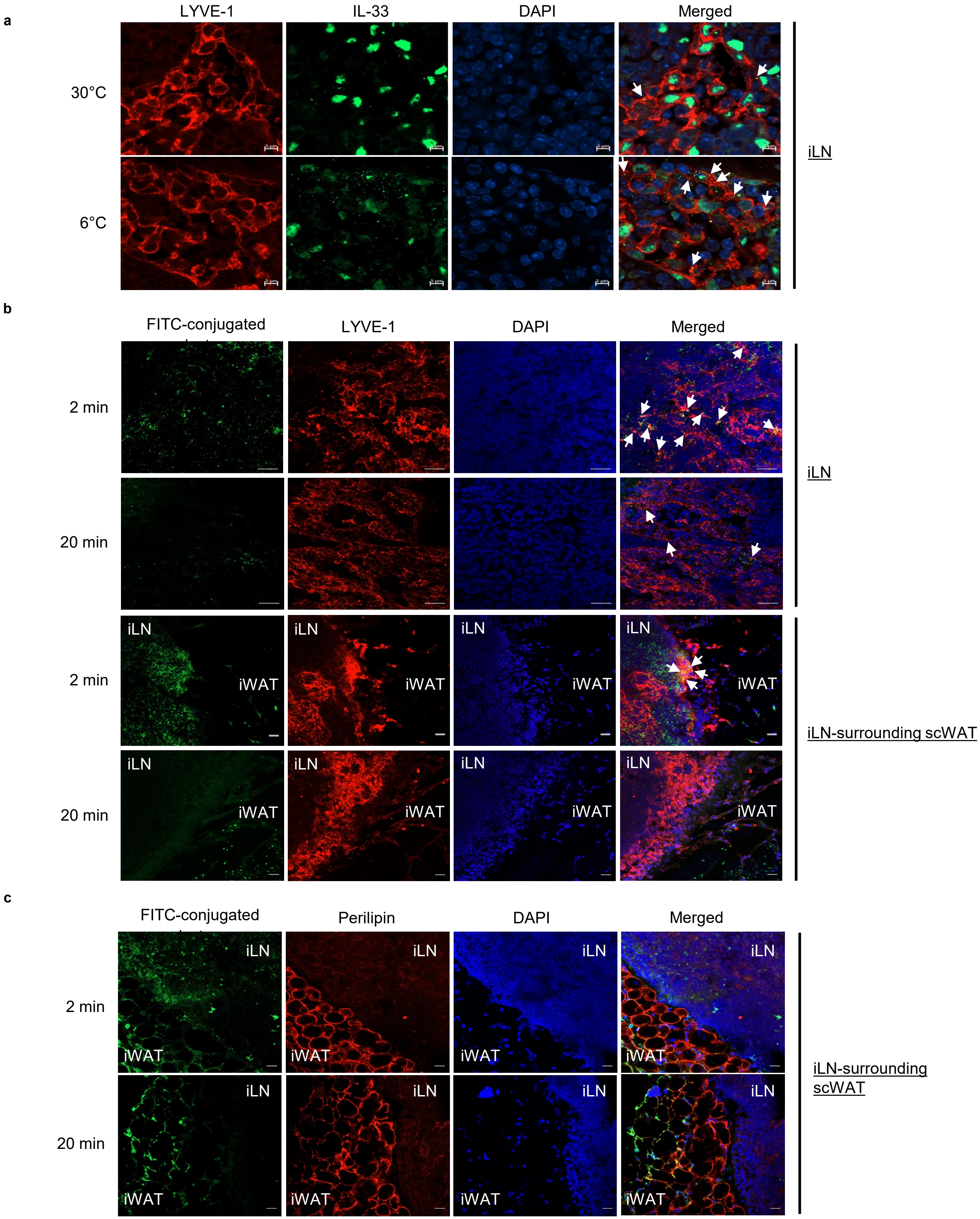

Fig. S8. Related to Fig. 3. IL-33 exits iLN via lymphatic vessels. (a) Representative confocal images of immunofluorescence staining for the lymphatic vessel marker LYVE-1 (red) and IL-33 (green) under 30°C or 6°C in iLN. Scale bar, 5 μm. **(b-c)** Representative confocal images for co-localization of dextran (green), LYVE-1 (red) (b) and perilipin (red) (c) in iLN and its surrounding inguinal scWAT. FITC-conjugated dextran (40 kDa) was directly injected into iLN. Mice were sacrificed after injection for 2 min and 20 min, respectively, followed by immunostaining and confocal microscopic analysis. Scale bar, 20 μm. Each experiment was repeated independently for three times.

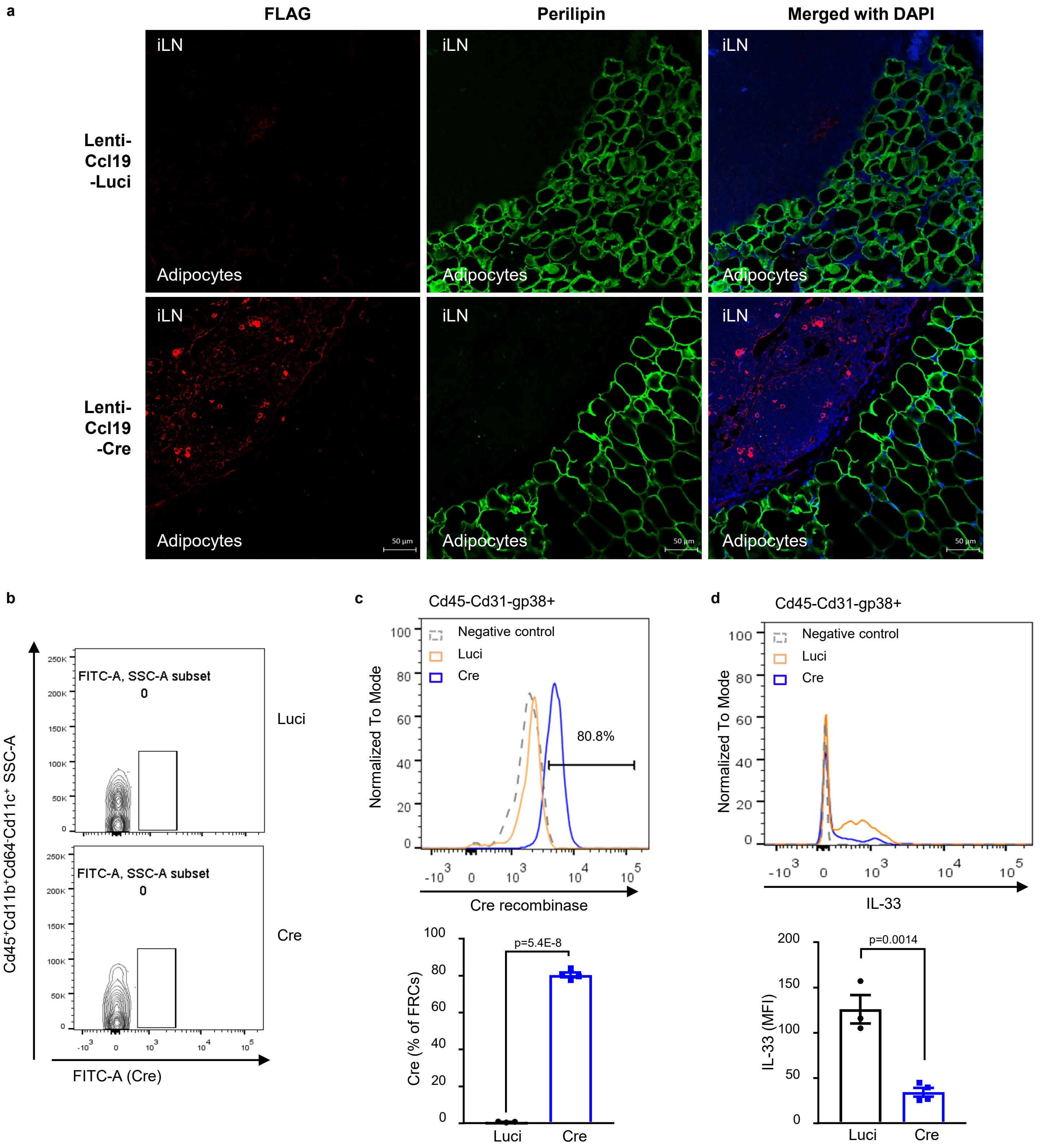

Fig. S9. Related to Fig. 3. The specificity and transduction efficacy of Ccl19 promoter-driven expression of FLAG-tagged Cre recombinase in fibroblastic reticular cells in iLNs. Lentivirus encoding FLAG-tagged Cre and luciferase (Lenti-Ccl19-Cre) or luciferase only (Lenti-Ccl19-Luci) driven by the Ccl19 promoter was directly injected into iLNs (7.5×10^6 TU per side) of eight-week-old male $IL33^{fl/fl}$ mice. 7 days after injection, mice were housed at 30°C for 3 weeks followed by 2-day cold exposure (6°C) or continued to be housed at 30°C for another 2 days. **(a)** Representative confocal images of iLN and scWAT sections co-stained with antibodies against FLAG tag (red) and perilipin (green). Scale bar, 50 μm. **(b)** Representative contour plots of dendritic cells (Cd45+Cd11b+Cd64-Cd11c+) as determined by flow cytometric analysis. **(c-d)** Representative histogram overlays of flow cytometric analysis for Cre recombinase-positive (top) and quantification of the percentage of Cre recombinase (bottom) (c) and IL-33 (top) and quantification of median fluorescence intensity (MFI) for IL-33 (bottom) (d) in FRCs (Cd45-Cd31-gp38+Cre+) isolated from iLNs. For Luci (n = 3) or Cre (n =4) are biologically independent replicates. Data are presented as mean ± SEM. Statistical data were assessed using unpaired two-tailed Student's t test (**c-d**). All the p values were two-sided. Source data are available as a Source Data file**.**

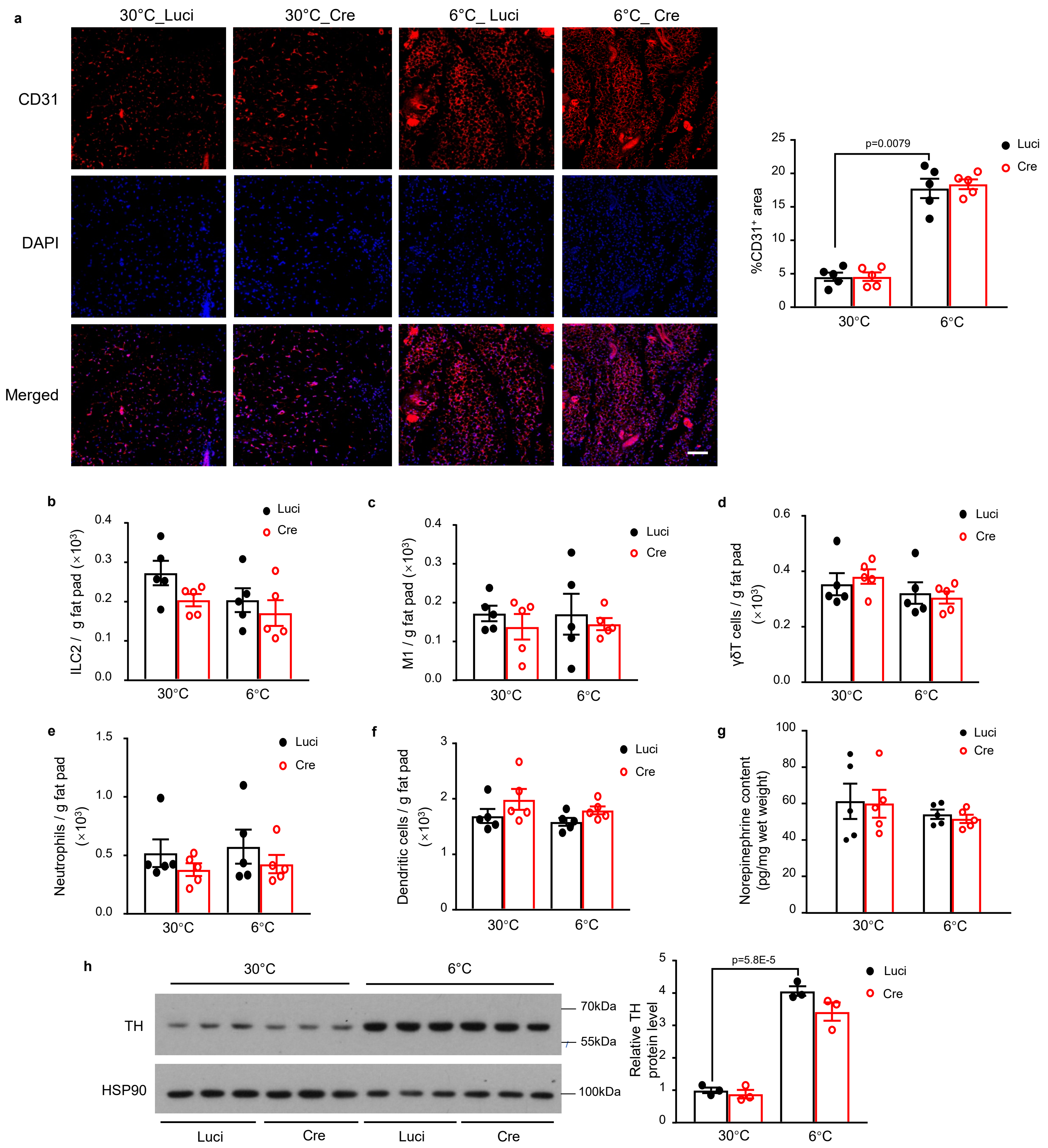

Fig. S10. Related to Fig. 3. Effects of lentivirus-mediated conditional knockout of IL-33 on vascularization, recruitment of immune cells and sympathetic innervation in scWAT. (a) Representative confocal images of immunofluorescence staining for blood vessel marker CD31 (red) in scWAT of mice injected with Lenti-Ccl19-Cre or Lenti-Ccl19-Luci under 30°C or 6°C for 2 days. The right panel is the quantification of $CD31^{+}$ signals (n = 5). Scale bar, 50 µm. **(b-f)** Absolute numbers of ILC2s (b), M1 macrophages (c), γδT cells (d), neutrophils (e) and dendritic cells (f) in scWAT (n = 5). **(g)** LC/MS-based measurement of norepinephrine content in iLN-surrounding inguinal scWAT (n = 5). **(h)** Western blot analysis for TH in scWAT (top) and densitometric quantification for the relative abundance of TH normalized with HSP90 (bottom) (n = 3). All samples are biologically independent replicates. Data are presented as mean ± SEM. Statistical data were assessed using Mann-Whitney U test **(a)** or unpaired two-tailed Student's t test (**h**). All the p values were two-sided. Source data are available as a Source Data file. kDa, relative molecular weight in kilodalton.

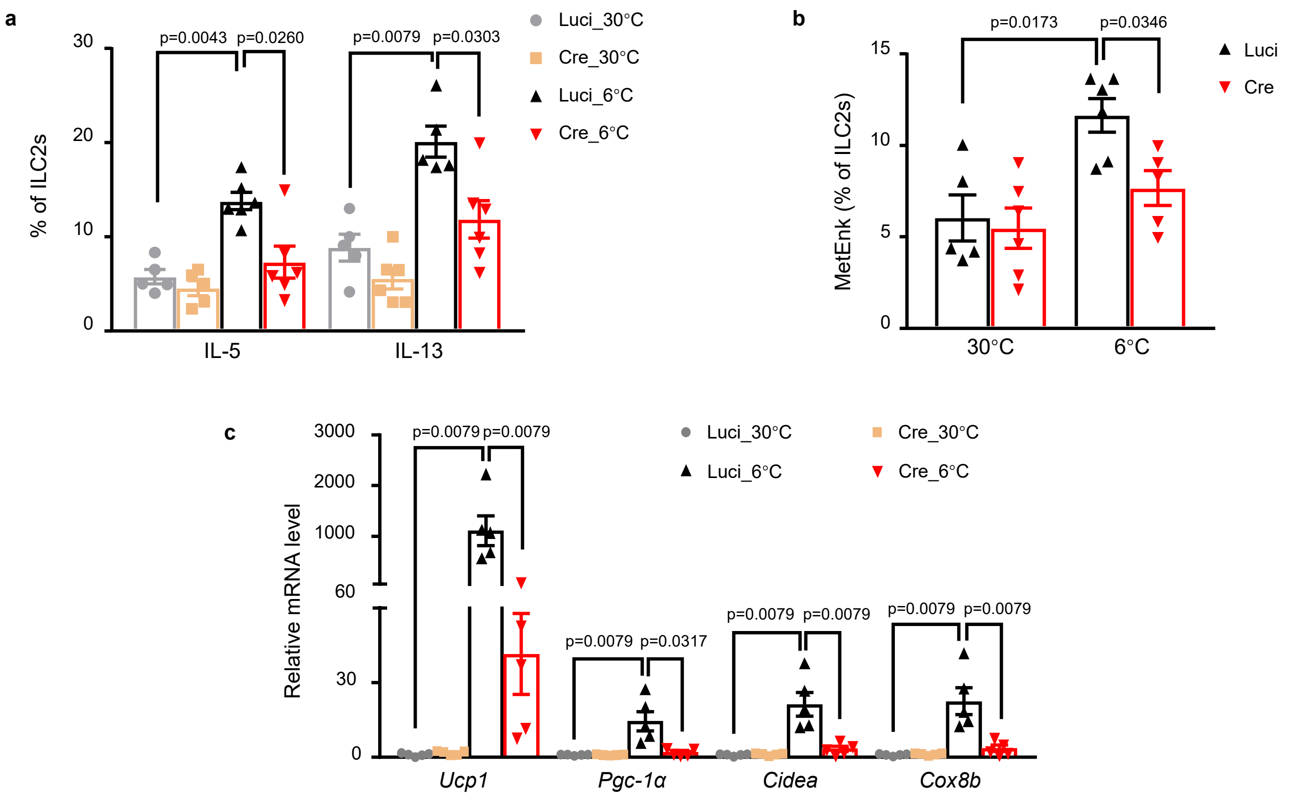

Fig. S11. Related to Fig. 3. Unilateral injection of lentivirus encoding Cre recombinase has no impact on the contralateral iLN and scWAT. (a-c) IL33$^{fl/fl}$ mice with unilateral injection of lentivirus encoding Ccl19-driven FLAG-tagged Cre and luciferase (Lenti-Ccl19-Cre) or luciferase only (Lenti-Ccl19-Luci) into iLNs (7.5×10^6 TU per side) were exposed to 6°C or 30°C for 2 days. **(a-b)** Flow cytometric quantification for IL-5 and IL-13 (a) (n = 5 for Luci_30°C and Cre_30°C or n = 6 for Luci_6°C and Cre_6°C) and MetENK (b) (n = 5 for Luci_30°C and Cre_6°C or n = 6 for Cre_30°C and Luci_6°C) in ILC2s. **(c)** mRNA level of thermogenic genes in scWAT (n = 5). All samples are biologically independent replicates. Data are presented as mean $\pm$ SEM. Statistical data were assessed using Mann-Whitney U test **(a-c).** All the p values were two-sided. Source data are available as a Source Data file**.**

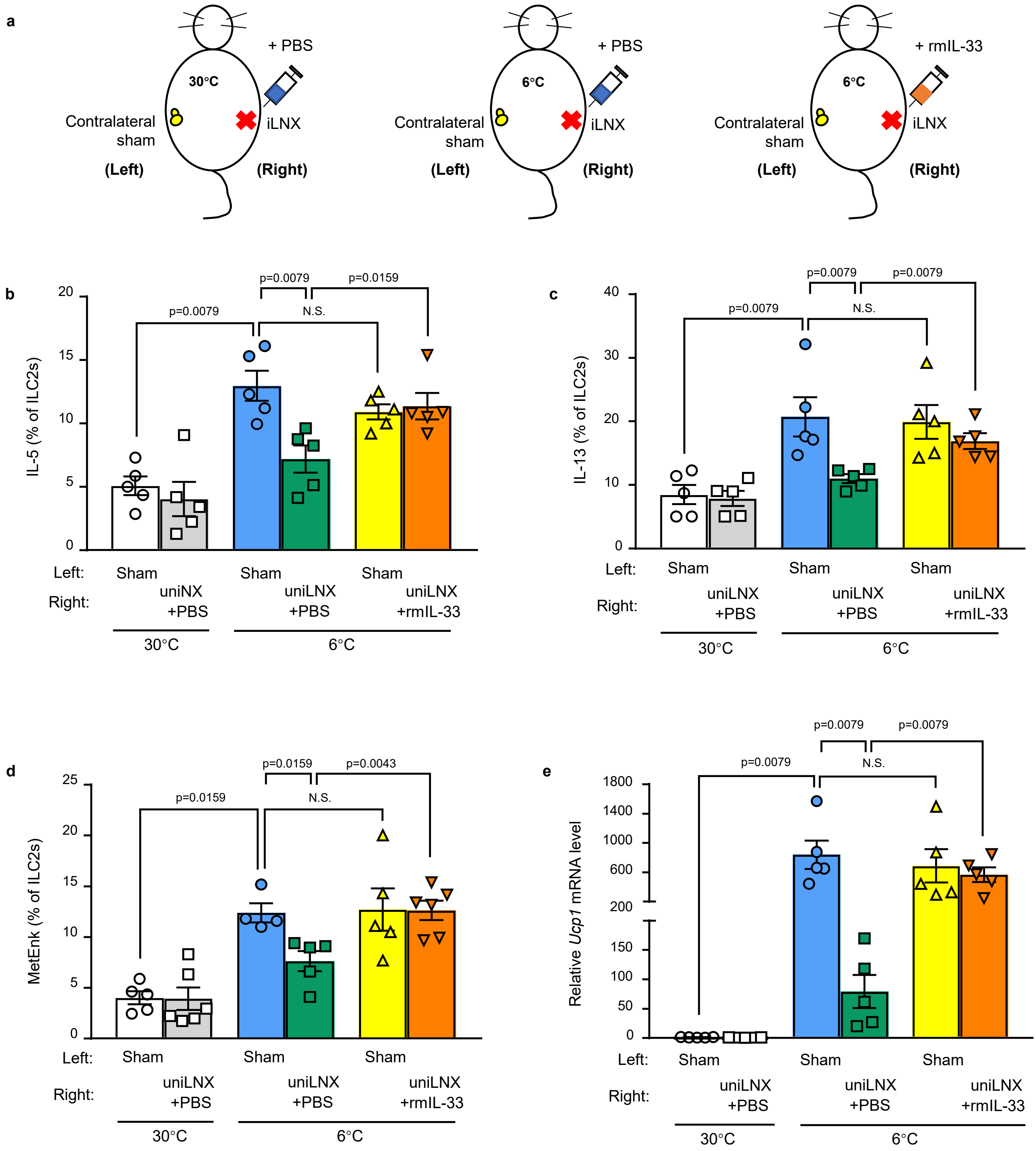

Fig. S12. Related to Fig. 4. Unilateral treatment of rmIL-33 in the side of iLN depletion does not affect ILC2 activation and beiging in the contralateral scWAT control. Eight-week-old male C57BL/6N mice were subjected to uniLNX and sham operation on the contralateral side. After surgery, the mice were housed at 30°C for 3 weeks, and subsequently subjected to unilateral injection of 250 ng rmIL-33 or PBS into the uniLNX side of scWAT for 4 consecutive days. Mice were then exposed to 6°C for another 2 days after the first two injections. **(a)** Schematic diagram showing experimental design. **(b-d)** Flow cytometric analysis for the quantification of IL-5 (b) (n = 5), IL-13 (c) (n = 5) and MetENK (d) (n = 5 for Sham _ 30°C and Sham_ 6°C; n = 6 for uniLNX+PBS_30°C and uniLNX+rmIL-33_6°C; n = 5 for iLNX_PBS_6°C) positive cells in activated ILC2s from scWAT. **(e)** Real-time PCR analysis of the mRNA expression of UCP1 (n = 5). All samples are biologically independent replicates. Data are presented as mean ± SEM. Statistical data were assessed using Mann-Whitney U test **(b-e).** All the p values were two-sided. Source data are available as a Source Data file. kDa, relative molecular weight in kilodalton; N.S., not significant.

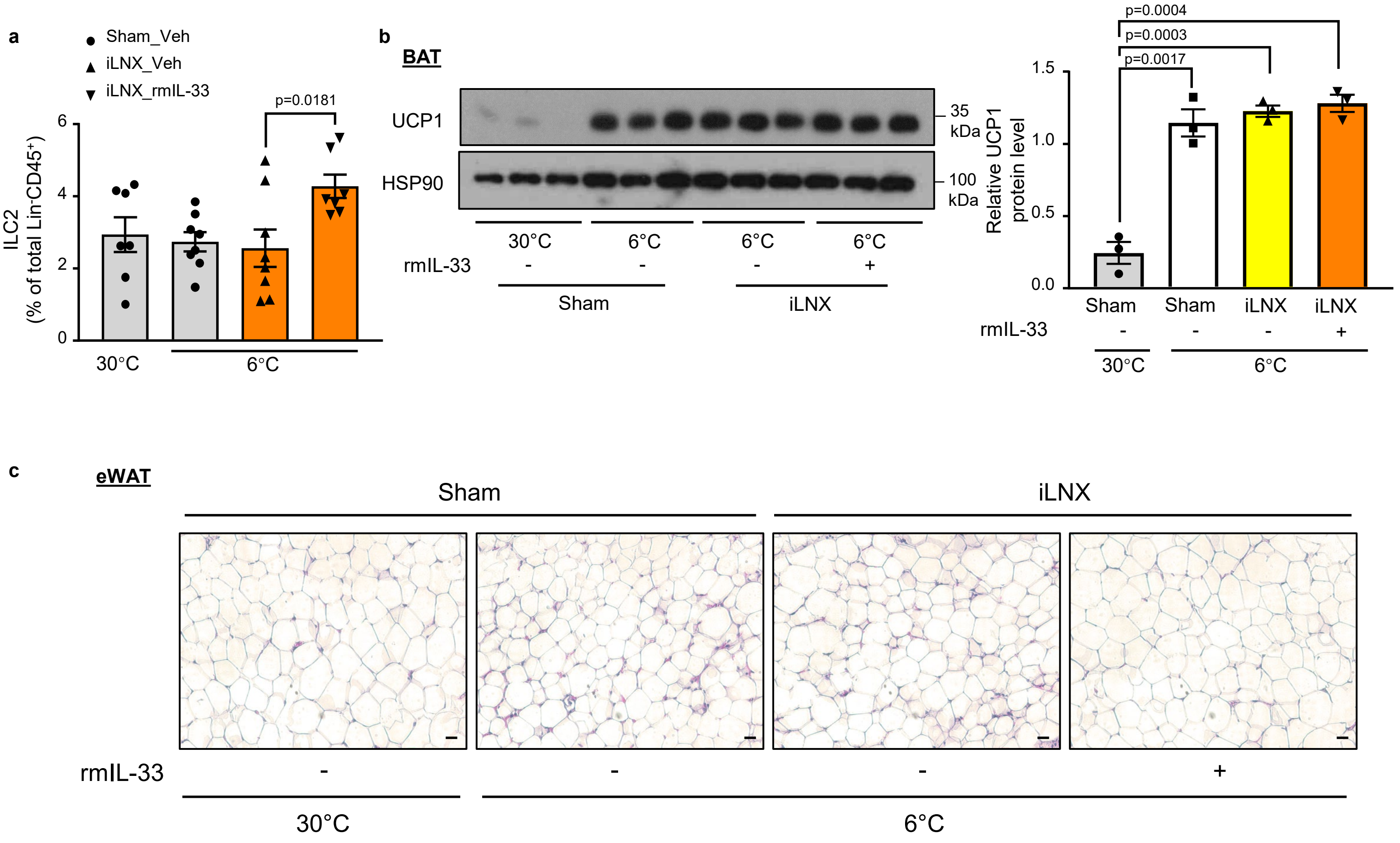

Fig. S13. Related to Fig. 4. Administration of rmIL-33 into scWAT does not affect UCP1 expression in BAT and morphology of eWAT in mice. Eight-week-old iLNX and sham-operated male C57BL/6N mice housed at 30°C for 3 weeks were subjected to direct injection of 250 ng rmIL-33 or PBS into the scWAT for 4 consecutive days. Mice were then subjected to 6°C or continued to be housed at 30°C for 2 days after the first two injections**.** **(a)** Flow cytometric analysis of the percentage of ILC2s in SVF of scWAT. (n=7 for Sham_Veh_30°C and iLNX_rmIL-33_6°C or n =8 for Sham_Veh_6°C and iLNX_iLNX_6°C group). **(b)** UCP1 protein level in BAT was determined by Western blot analysis. The right panel is the densitometric quantification of the relative abundance of UCP1 normalized to HSP90 (n = 3). **(c)** Representative images of eWAT stained with H&E. Scale bar, 20 μm. Each experiment was repeated independently for three times. All samples are biologically independent replicates. Data are presented as mean ± SEM. Statistical data were assessed using unpaired two-tailed Student's t test **(a-b).** All the p values were two-sided. Source data are available as a Source Data file**.** kDa, relative molecular weight in kilodalton.

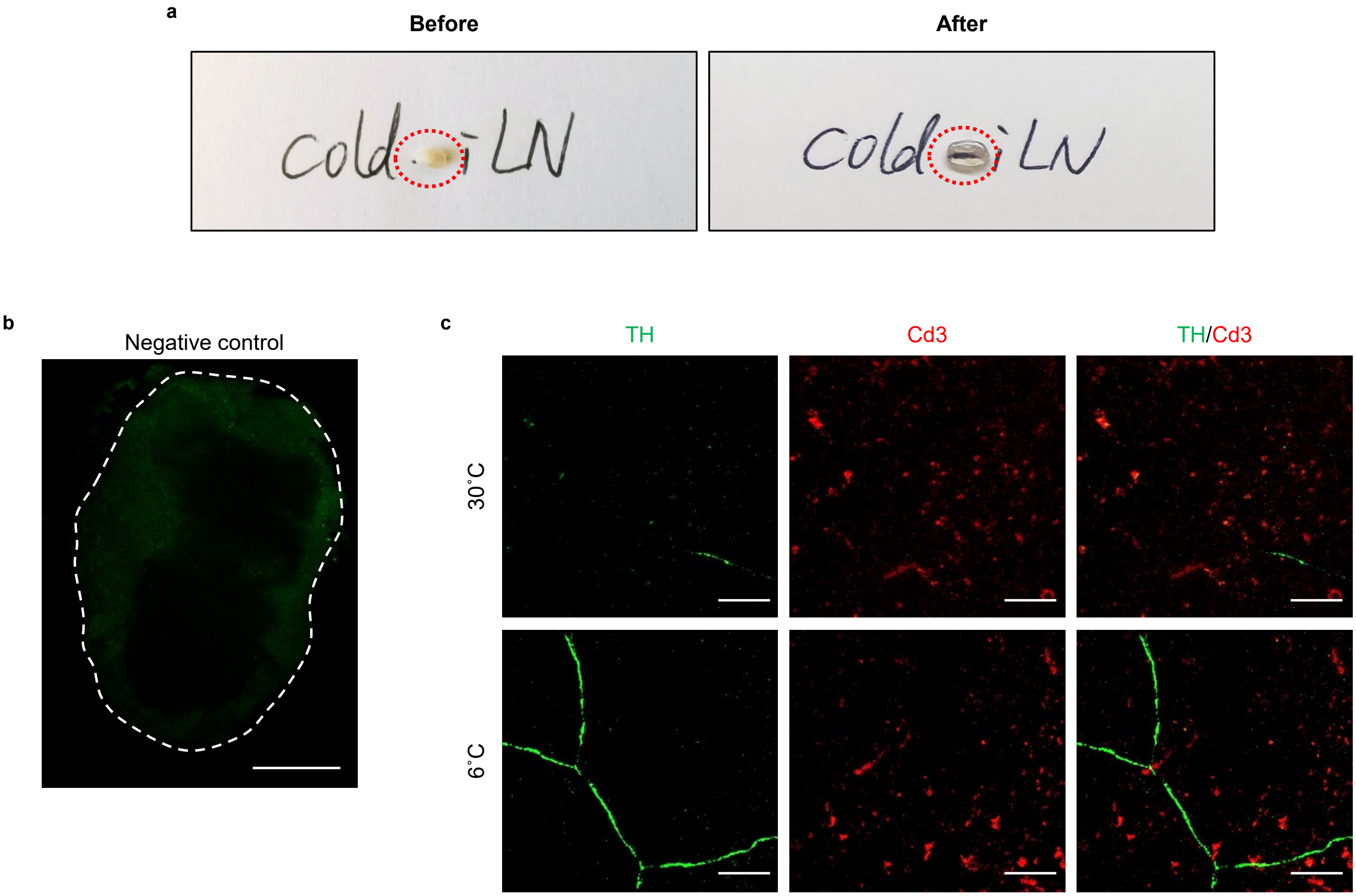

Fig. S14. Related to Fig. 5. Cd3-positive lymphocytes in iLN do not express TH. iLN was isolated from 8-week-old male C57BL/6N mice housed at 30°C for three weeks before subjecting to cold exposure (6°C) or continued to be housed at 30°C for 2 days. **(a)** iLN of mice before (left) and after (right) tissue optical clearing. **(b)** Negative control (stained with non-immune rabbit IgG) of whole-mount imaging to show autofluorescence of iLN. Scale bar, 500 μm. **(c)** Immunofluorescence staining of TH (green) and Cd3 (red) in iLN. Scale bar, 50 μm. Each experiment was repeated independently for three times.

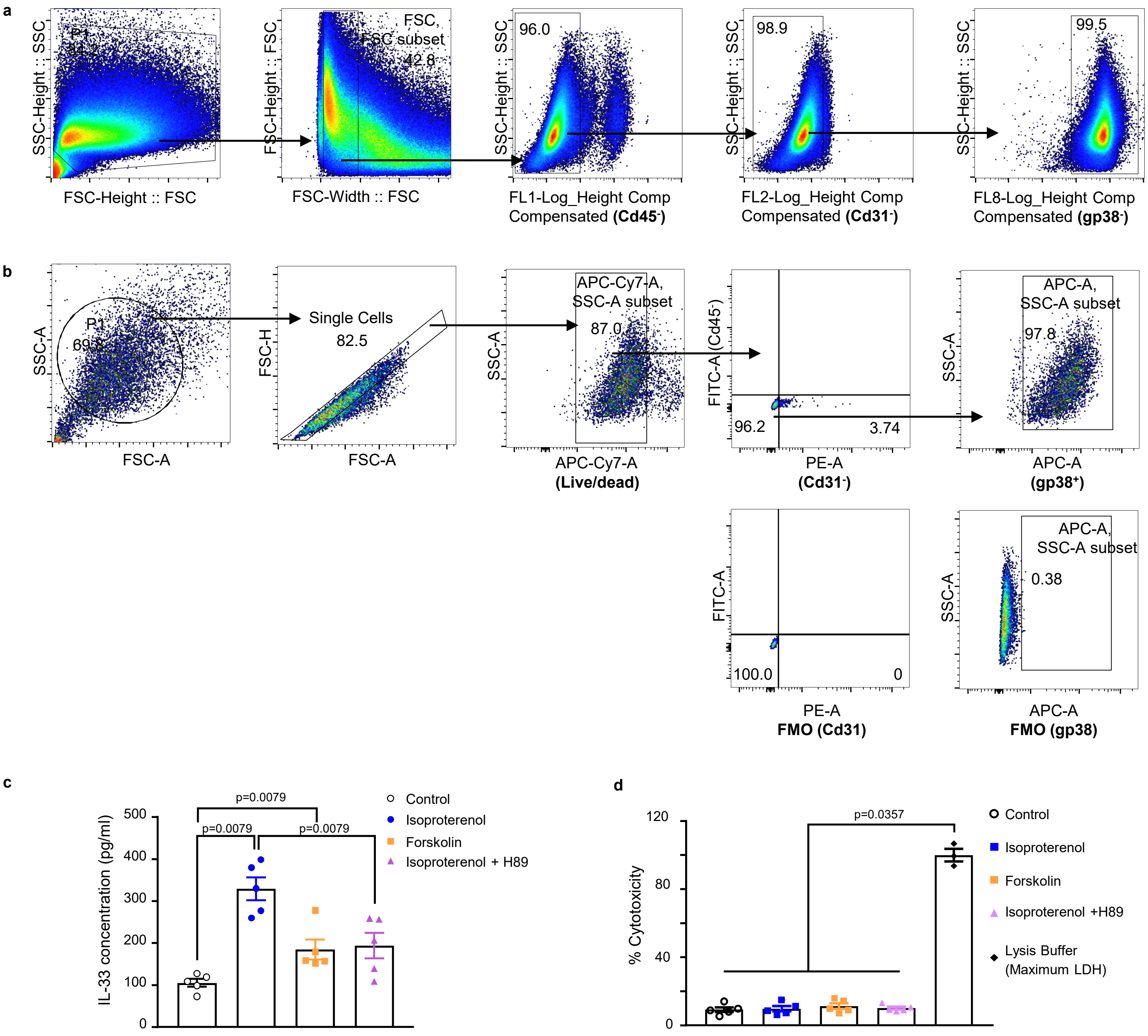

Fig. S15. Related to Fig. 5. LN FRCs secrete IL-33 in response to β adrenergic stimulation. **(a-c)** FRCs were isolated from peripheral iLNs of 8-week-old male C57BL/6N mice for culturing and then subjected to cell sorting. **(a-b)** Gating strategies to purify (a) and verify (b) cell population of FRCs ($Cd45^{-}Cd31^{-}gp38^{+}$) in cultured cells. **(c-d)** ELISA analysis of IL-33 (c) (n = 5) and (d) quantification of lactate dehydrogenase (LDH) in the extracellular medium of FRCs treated with isoproterenol (10 μM), forskolin (5 μM) and PKA inhibitor H89 (5 μM) for 8 hours (n = 5 for Control, Isoproterenol, Forskolin and Isoproterenol+H89; n = 3 for Lysis Buffer_Maximum LDH). All samples are biologically independent replicates. Data are presented as mean ± SEM. Statistical data were assessed using Mann-Whitney U test **(c-d).** All the p values were two-sided. Source data are available as a Source Data file**.**

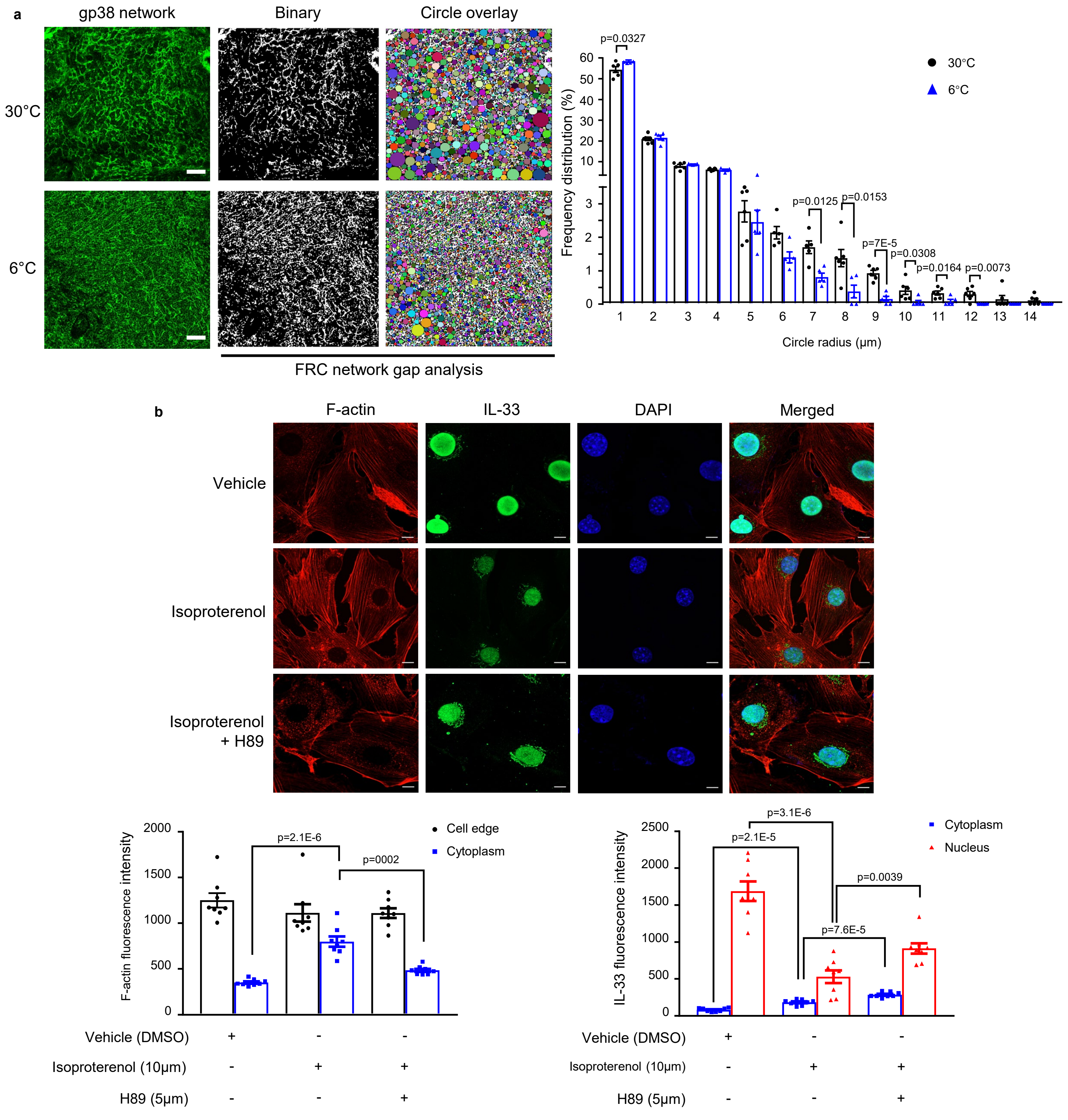

Fig. S16. Related to Fig. 5. Cold-induced release of IL-33 from FRCs in iLN is related to cell contraction. iLN was isolated from 8-week-old male C57BL/6N mice housed at 30°C for three weeks before subjecting to cold exposure (6°C) or continued to be housed at 30°C for 2 days. **(a)** gp38 staining (green, FRCs) of iLN sections were converted to binary and gaps (coloured circles overlay) within the FRC network for gap analysis (left) and quantification of circle radius (right) (n = 6 for 30°C or n =5 for 6°C). Scale bar, 50 μm. **(b)** Cultured FRCs were treated with isoproterenol (10 μM), or isoproterenol and PKA inhibitor H89 (5 μM) for 8 hours. Representative confocal images of immunofluorescence staining for the actomyosin marker F-actin (red) and IL-33 (green) were shown. The lower panels are average fluorescence intensity at cell edge, cytoplasm and nucleus qualified as described in the Methods section (n = 8). Scale bar, 20 μm. All samples are biologically independent replicates. Data are presented as mean ± SEM. Statistical data were assessed using unpaired Student's t test **(a-b).** All the p values were two-sided. Source data are available as a Source Data file**.**

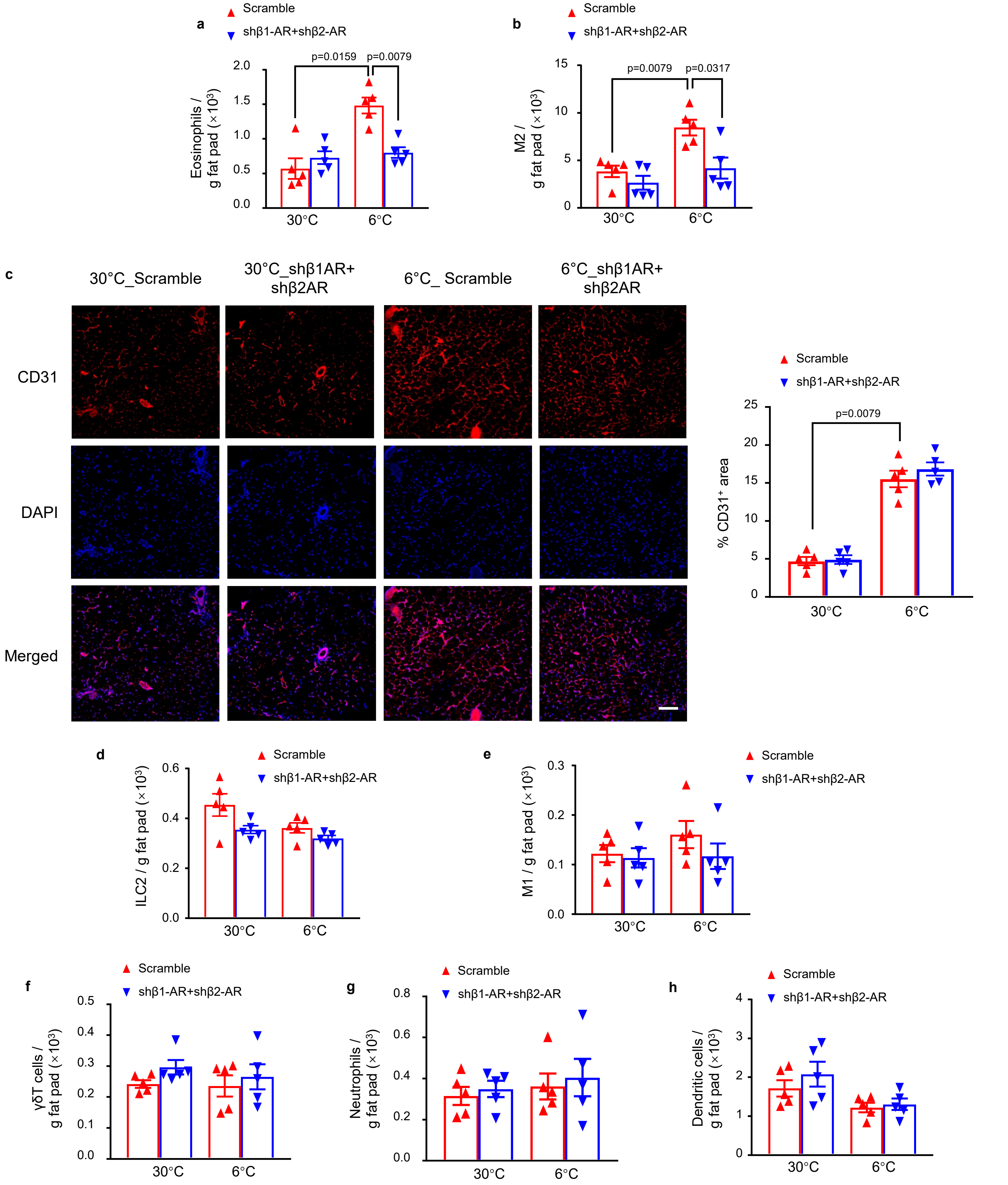

Fig. S17. Related to Fig. 5. Effects of lentivirus-mediated conditional knockout of β1- and β2-AR on vascularization and recruitment of immune cells in scWAT. (a-b) Flow cytometric analysis of absolute numbers of eosinophils (a) and M2 macrophages (b). **(c)** Representative confocal images of immunofluorescence staining for the blood vessel marker CD31 (red) in scWAT of mice with Lenti-Ccl19-shβ1-AR+shβ2-AR or Lenti-Ccl19-scramble injection under 30°C or 6°C for 2 days. The right panel is the quantification of $CD31^+$ signals. Scale bar, 50 μm. **(d-h)** Absolute numbers of ILC2s (d), M1 macrophages (e), γδT cells (f), neutrophils (g) and dendritic cells (h) in scWAT. All samples are n =5 biologically independent replicates. Data are presented as mean ± SEM. Statistical data were assessed using Mann-Whitney U test **(a-c).** All the p values were two-sided. Source data are available as a Source Data file.

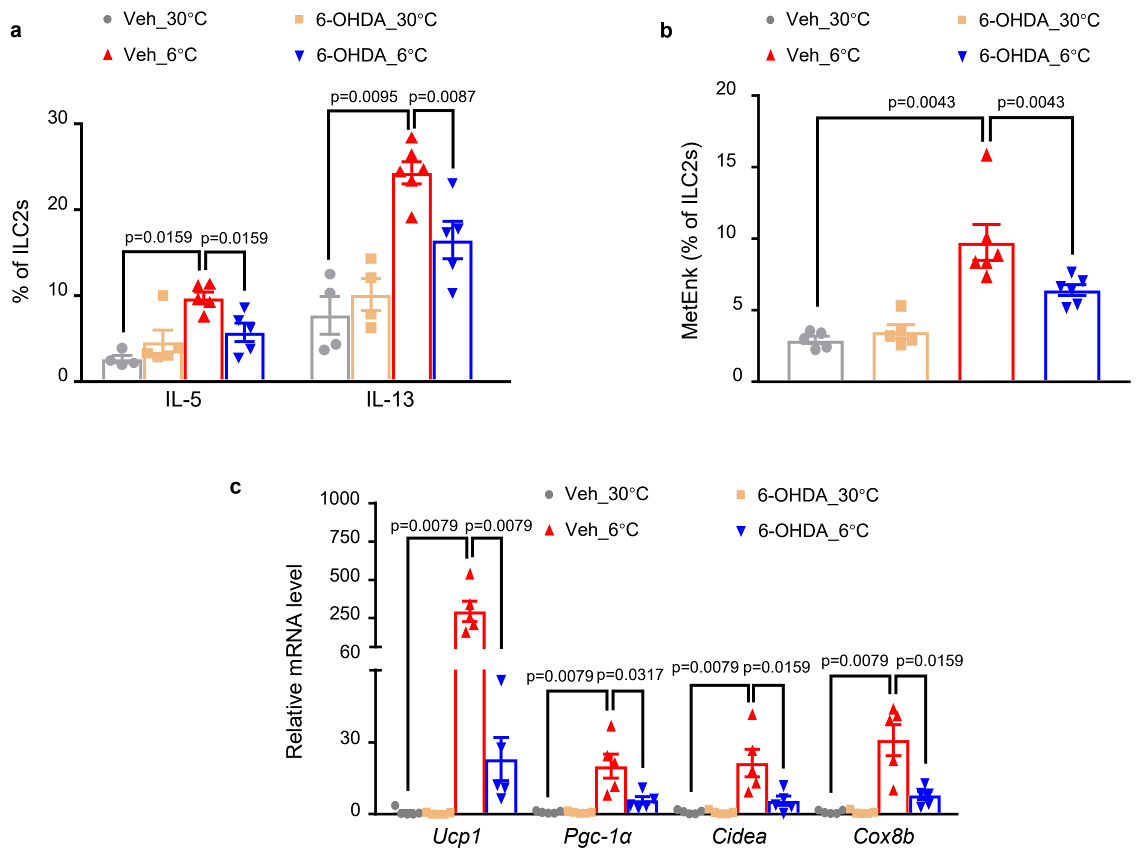

Fig. S18. Related to Fig. 6. Unilateral denervation of iLN has no effect on the contralateral control side. Eight-week-old male C57/BL6N mice were subjected to unilateral sympathetic denervation of iLN by unilateral local injection of 6-OHDA (9 mg/ml in 0.15M NaCl) or 0.15M NaCl as vehicle control (Veh) directly into iLN of the same mouse. Afterwards, mice were housed at 30°C for 3 weeks followed by cold exposure (6°C) or continued to be housed at 30°C for another 2 days. **(a-b)** Quantification of percentage of IL-5- and IL-13- (a) (IL-5: n = 4 for Veh_30°C or n =5 for 6-OHDA_30°C, Veh_6°C and 6-OHDA_6°C; IL-13: n = 4 for Veh_30°C and 6-OHDA_30°C or n = 6 for Veh_6°C or n = 5 for 6-OHDA_6°C) and MetENK- (b) (n = 5 for Veh_30°C and 6-OHDA_30°C; n = 6 for Veh_6°C and 6-OHDA_6°C) positive cells in activated ILC2s from sWAT. **(c)** mRNA level of thermogenic genes in scWAT (n = 5). All samples are biologically independent replicates. Data are presented as mean ± SEM. Statistical data were assessed using Mann-Whitney U test **(a-c).** All the p values were two-sided. Source data are available as a Source Data file**.**

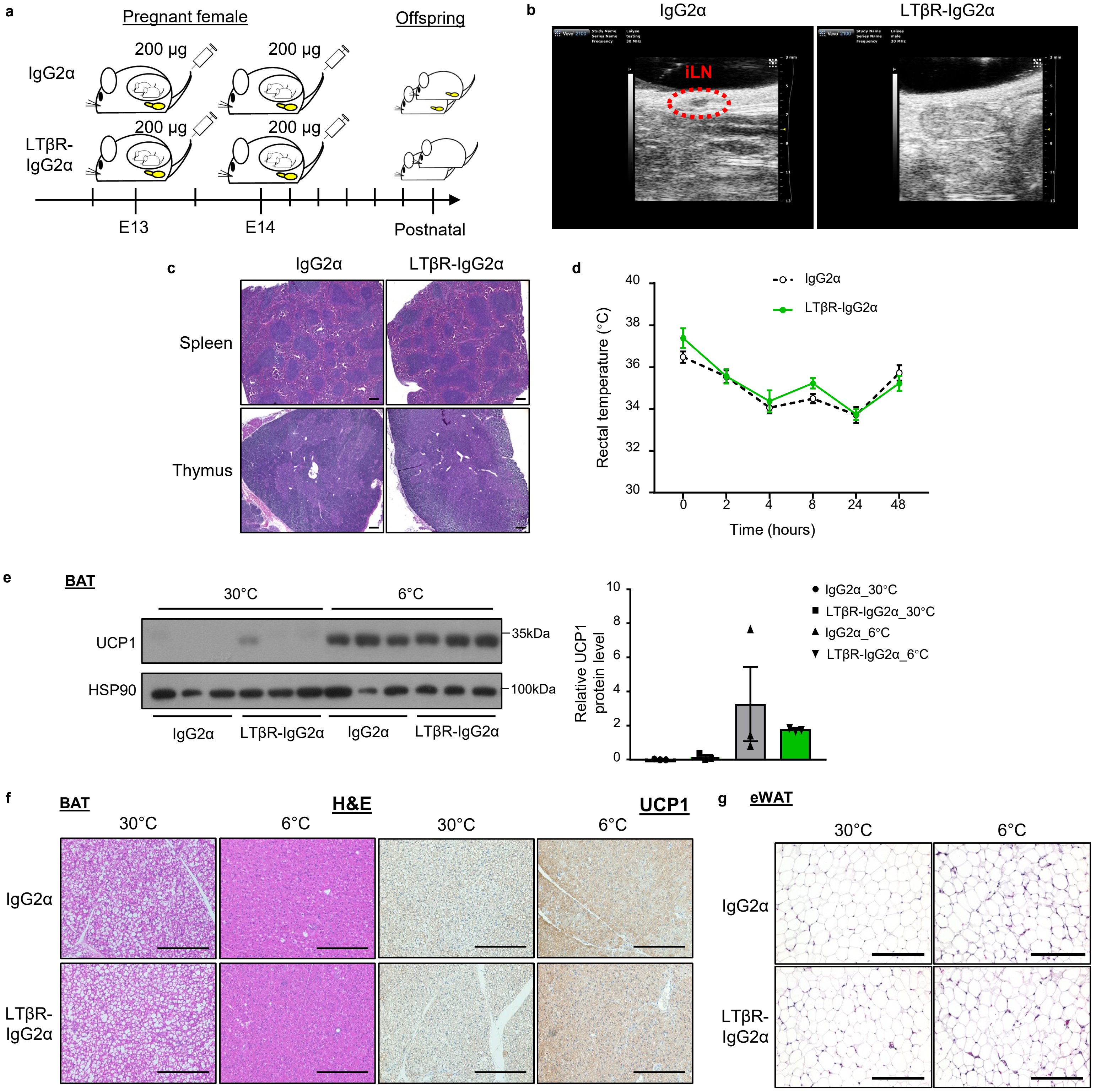

Fig. S19. Related to Fig. 7. LTβR-IgG2α-treated iLN-free offsprings show no obvious changes in cold-induced thermogenesis of classical BAT and morphology of eWAT. Pregnant female C57BL/6N mice were intravenously injected with LTβR-IgG2α (200 μg) or IgG2α isotype on E13 and E14 to generate iLNs-depleted progeny mice. **(a)** Schematic diagram for the generation of iLNs-depleted offsprings. **(b)** Representative sonograms showing the presence and absence of iLN in 8-week-old male IgG2α- and LTβR-IgG2α-treated progeny mice respectively. **(c)** Representative images of spleen and thymus sections stained with H&E. Scale bar, 200 μm. **(d)** Rectal temperature of mice was measured at different time points during 2 days of 6°C (n = 5). **(e)** Western blot analysis of UCP1 protein expression in BAT (left) and densitometric analysis for the relative abundance of UCP1 normalized with HSP90 (right) (n = 3). **(f)** Representative images of histological sections stained with H&E or an anti-UCP1 antibody in BAT. Scale bar, 200 μm. **(g)** Representative images of H&E-stained sections in eWAT. Scale bar, 200 μm. All samples are biologically independent replicates. Data are presented as mean ± SEM. Source data are available as a Source Data file**.** kDa, relative molecular weight in kilodalton.

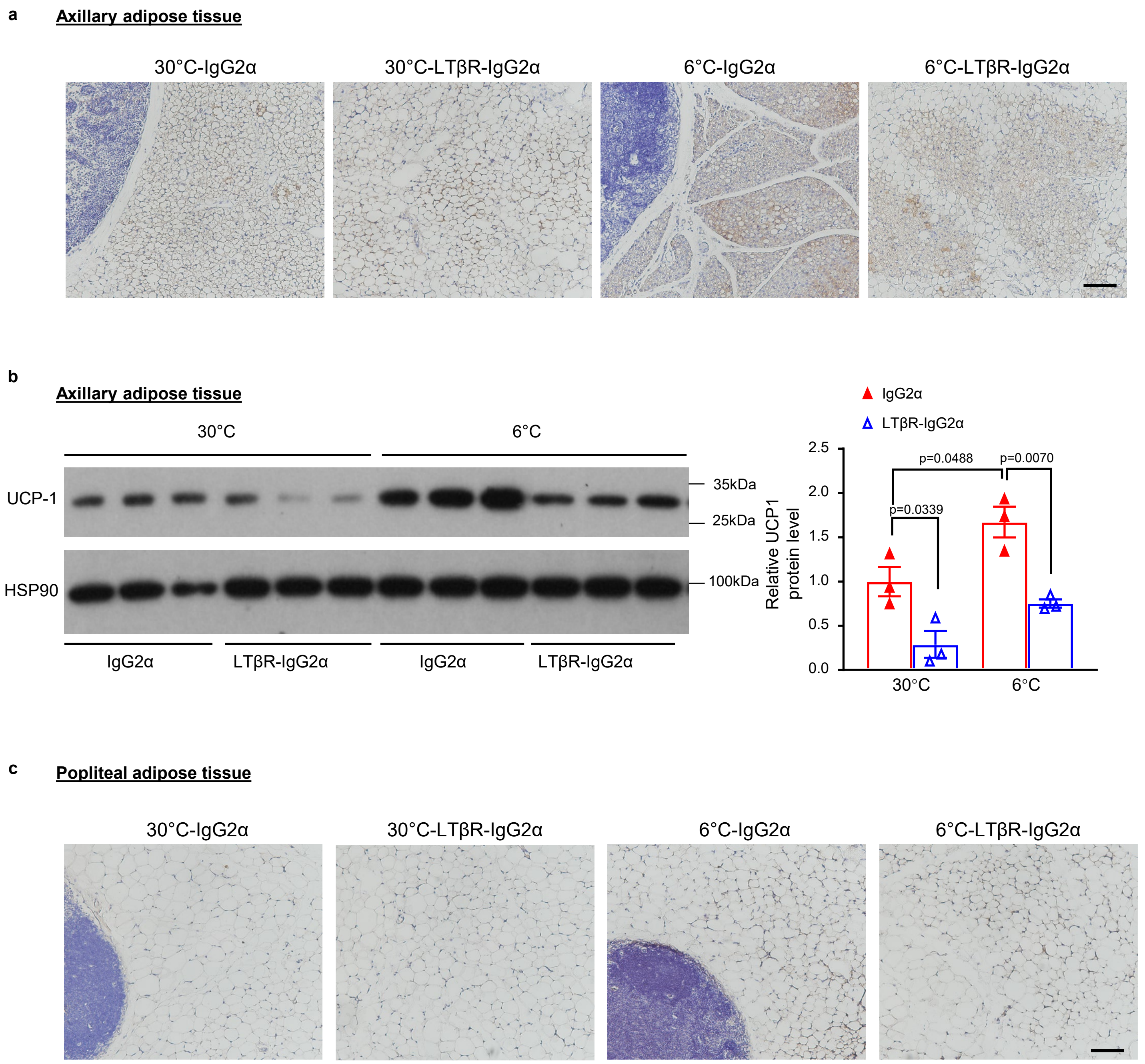

Fig. S20. Related to Fig. 7. Roles of axillary and popliteal lymph nodes on cold-induced beiging in their surrounding adipose tissue. Pregnant female C57BL/6N mice were intravenously injected with LTβR-IgG2α (200 μg) or IgG2α isotype on E13 and E14 to generate iLNs-depleted progeny mice. Eight-week-old LTβR-IgG2α-treated mice and their controls were housed at 30°C for three weeks before subjecting to cold exposure at 6°C for 2 days. **(a)** DAB staining of UCP1 in axillary adipose tissue. Scale bar, 100 μm. **(b)** Western blot analysis of UCP1 in axillary adipose tissue. The right panel is the densitometric analysis for the relative abundance of UCP1 normalized with HSP90 (n = 3 biologically independent replicates). Note that UCP1 protein in popliteal adipose tissues is not detectable by Western blot even after cold exposure. **(c)** DAB staining of UCP1 in popliteal adipose tissue. Scale bar, 100 μm. Statistical data were assessed using unpaired two-tailed Student's t test **(b).** All the p values were two-sided. Source data are available as a Source Data file. kDa, relative molecular weight in kilodalton. .

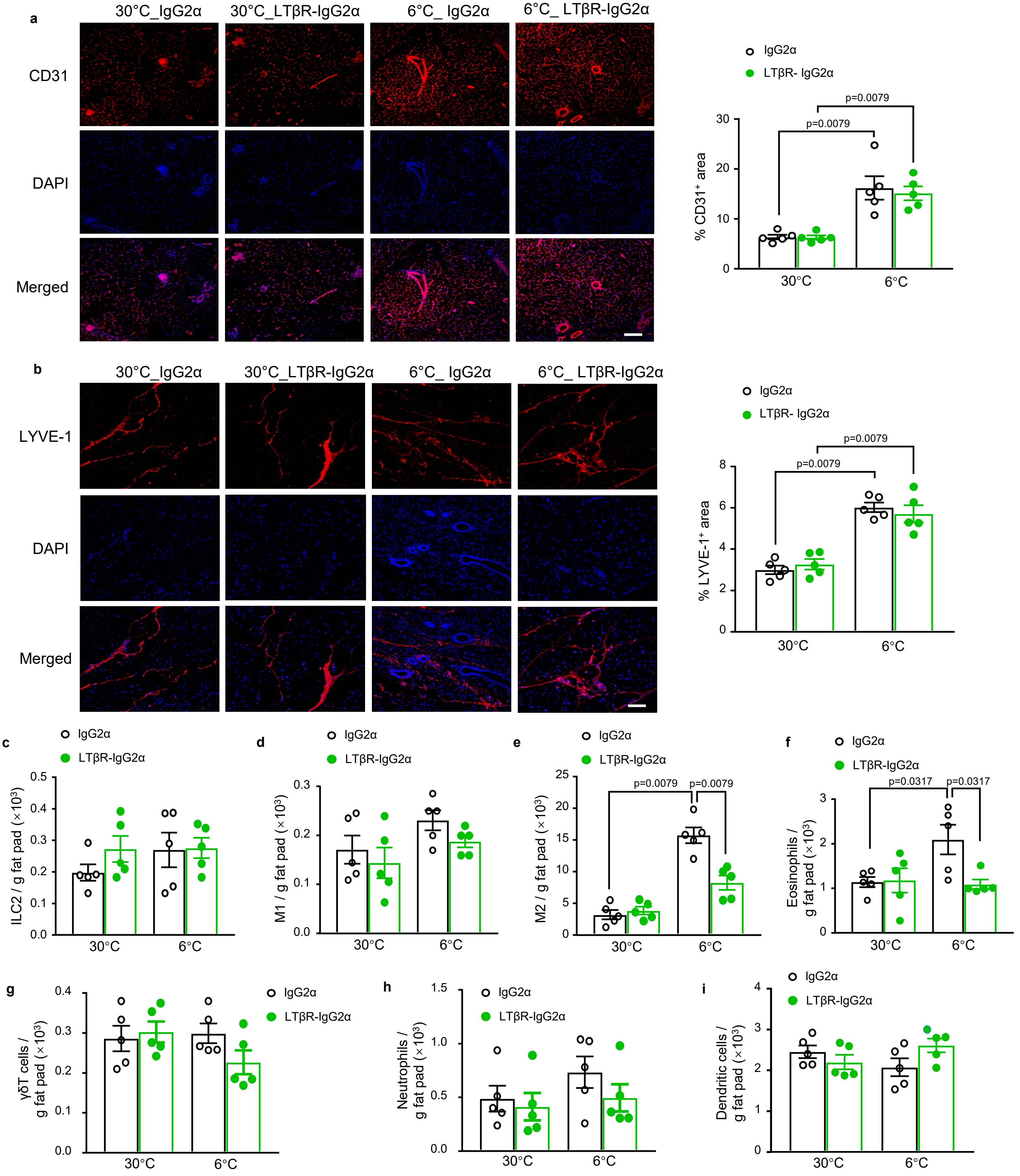

Fig. S21. Related to Fig. 7. Effects of pharmacological depletion of iLN on lymphatic and blood vessels, and recruitment of immune cells in iLN-surrounding scWAT. (a-b) Representative confocal images of immunofluorescence staining for the blood vessel marker CD31 (a, red) or lymphatic vessel marker LYVE-1 (b, red) in scWAT of LTβR-IgG2α-treated mice and the IgG2α-treated control mice under 30°C or 6°C for 2 days. The right panels are the quantification of CD31⁺ or LYVE-1⁺ signals, respectively (n = 5). Scale bar, 50 μm. **(c-i)** Flow cytometric analysis of absolute numbers of ILC2s (c), M1 and M2 macrophages (d, e), eosinophils (f), γδT cells (g), neutrophils (h) and dendritic cells (i) in entire scWAT (n = 5). All samples are biologically independent replicates. Data are presented as mean ± SEM. Statistical data were assessed using Mann-Whitney U test **(a-b, e-f).** All the p values were two-sided. Source data are available as a Source Data file**.**
