# Supplementary material for: Fibroblastic reticular cells in lymph node potentiate white adipose tissue beiging through neuro-immune crosstalk in male mice
Source: Nat Commun. 2023 Mar 3;14:1213. doi: 10.1038/s41467-023-36737-0 (PMC9984541; doi:10.1038/s41467-023-36737-0)
Supplement: Supplementary file 2 — Description of Additional Supplementary Files [file 41467_2023_36737_MOESM2_ESM.pdf]

Title: Supplementary Data 1

Description: The sequences of shRNAs and primers used for real-time PCR analysis.
