# Supplementary material for: Fibroblastic reticular cells in lymph node potentiate white adipose tissue beiging through neuro-immune crosstalk in male mice
Source: Nat Commun. 2023 Mar 3;14:1213. doi: 10.1038/s41467-023-36737-0 (PMC9984541; doi:10.1038/s41467-023-36737-0)
Supplement: Supplementary file 4 — Reporting Summary [file 41467_2023_36737_MOESM4_ESM.pdf]

nature portfolio

Corresponding author(s): Aimin Xu, Baile Wang

Last updated by author(s): Feb 14, 2023

Reporting Summary

Nature Portfolio wishes to improve the reproducibility of the work that we publish. This form provides structure for consistency and transparency in reporting. For further information on Nature Portfolio policies, see our Editorial Policies and the Editorial Policy Checklist.

Statistics

For all statistical analyses, confirm that the following items are present in the figure legend, table legend, main text, or Methods section.

n/a	Confirmed	
☐	☒	The exact sample size (*n*) for each experimental group/condition, given as a discrete number and unit of measurement
☐	☒	A statement on whether measurements were taken from distinct samples or whether the same sample was measured repeatedly
☐	☒	The statistical test(s) used AND whether they are one- or two-sided<br>*Only common tests should be described solely by name; describe more complex techniques in the Methods section.*
☒	☐	A description of all covariates tested
☒	☐	A description of any assumptions or corrections, such as tests of normality and adjustment for multiple comparisons
☐	☒	A full description of the statistical parameters including central tendency (e.g. means) or other basic estimates (e.g. regression coefficient) AND variation (e.g. standard deviation) or associated estimates of uncertainty (e.g. confidence intervals)
☐	☒	For null hypothesis testing, the test statistic (e.g. *F*, *t*, *r*) with confidence intervals, effect sizes, degrees of freedom and *P* value noted<br>*Give P values as exact values whenever suitable.*
☒	☐	For Bayesian analysis, information on the choice of priors and Markov chain Monte Carlo settings
☒	☐	For hierarchical and complex designs, identification of the appropriate level for tests and full reporting of outcomes
☒	☐	Estimates of effect sizes (e.g. Cohen's *d*, Pearson's *r*), indicating how they were calculated

Our web collection on statistics for biologists contains articles on many of the points above.

Software and code

Policy information about availability of computer code

Data collection	BD LSRFortessa Cell Analyzer and MoFlo Optical Bench Sorter (Beckman Coulter) were used to collect flow cytometry data. IVIS in vivo imaging system (Perkin Elmer, Xenogen IVIS 124262) was used to collect images showing the localization and intensity of luciferase expression in mice. Basal oxygen consumption rate of adipose tissue explants was collected by Seahorse XFe24 extracellular flux analyzer (Agilent Technologies). Agilent 6460 Triple Quadrupole LC/MS system (Agilent Technologies) was used to collect norepinephrine content. Confocal images were collected using Zeiss ZEN software (version Blue 3.1). Body surface temperatures were measured using a FLIR T440 infrared camera, the quantification data were collected from FLIR Tools® analysis software. Real-time PCR data were collected by QuantStudio Real-Time PCR software v1.2.
Data analysis	All data were statistically analyzed in GraphPad Prism 7. Flow cytometry data were analyzed using FlowJo software version X.0.7.

For manuscripts utilizing custom algorithms or software that are central to the research but not yet described in published literature, software must be made available to editors and reviewers. We strongly encourage code deposition in a community repository (e.g. GitHub). See the Nature Portfolio guidelines for submitting code & software for further information.

Data

Policy information about availability of data

All manuscripts must include a data availability statement. This statement should provide the following information, where applicable:

- Accession codes, unique identifiers, or web links for publicly available datasets
- A description of any restrictions on data availability
- For clinical datasets or third party data, please ensure that the statement adheres to our policy

All data are available within the Article, Supplementary Information and Source Data file. Source data are provided with this paper. All materials generated in the current study are available from the corresponding authors on request.

Human research participants

Policy information about studies involving human research participants and Sex and Gender in Research.

Reporting on sex and gender	N/A
Population characteristics	N/A
Recruitment	N/A
Ethics oversight	N/A

Note that full information on the approval of the study protocol must also be provided in the manuscript.

Field-specific reporting

Please select the one below that is the best fit for your research. If you are not sure, read the appropriate sections before making your selection.

[x] Life sciences [ ] Behavioural & social sciences [ ] Ecological, evolutionary & environmental sciences

For a reference copy of the document with all sections, see nature.com/documents/nr-reporting-summary-flat.pdf

Life sciences study design

All studies must disclose on these points even when the disclosure is negative.

Sample size	Sample size determination is based on the previous experience to obtain significance and reproducibility (Huang Z, et al, Cell Metabolism, 2017, PMID: 28844880; Hui X, et al, Cell Metabolism, 2015, PMID: 26166748), as well as minimizing the number of animal used as required by the animal ethics committee. The sample size following common standards employing three or more biological replicates. All sample sizes are listed in each figure legend.
Data exclusions	No data was excluded.
Replication	All the experimental findings were repeated with three independent experiments. All attempts at replication were successful.
Randomization	All samples were randomly allocated in this study.
Blinding	The investigators were not blinded in this study because no bias could be made by the subject or the tester in the experiments performed.

Reporting for specific materials, systems and methods

We require information from authors about some types of materials, experimental systems and methods used in many studies. Here, indicate whether each material, system or method listed is relevant to your study. If you are not sure if a list item applies to your research, read the appropriate section before selecting a response.

Materials & experimental systems

n/a	Involved in the study
☐	☒ Antibodies
☒	☐ Eukaryotic cell lines
☒	☐ Palaeontology and archaeology
☐	☒ Animals and other organisms
☒	☐ Clinical data
☒	☐ Dual use research of concern

Methods

n/a	Involved in the study
☒	☐ ChIP-seq
☐	☒ Flow cytometry
☒	☐ MRI-based neuroimaging

Antibodies

Antibodies used

Antibodies for flow cytometry:
FITC lineage cocktail (anti-Cd3e, -Ly-6G, -Ly-6C, -Cd11b, -Cd45R/B220, -Ter-119) (1:200, clone 145-2C11, RB6-8C5, RA3-6B2, Ter-119, M1/70, Biolegend #133301)
Rat monoclonal antibody (mAb) against mouse Cd5-FITC (1:100, clone 53-7.3, Biolegend #100605)
Rat mAb against mouse Cd45-PerCP-Cyanine5.5 (1:100, clone 30-F11, eBioscience #45-0451-82)
Rat mAb against mouse IL33R- Phycoerythrin (PE) (1:100, clone U29-93, BD Biosciences #566311)
Rat mAb against mouse Cd127-PE-CyTM7 (1:100, clone SB/199, BD Biosciences #560733)
Rat mAb against mouse F4/80-PE (1:100, clone CI:A3-1, Abcam #ab105156)
Rat mAb against mouse Cd11b-BV421 (1:100, clone M1/70, BD Biosciences #562605)
Rat mAb against mouse Siglec F-Alexa Fluor® 647 (1:100, clone E50-2440, BD Biosciences #562680)
Rat mAb against mouse F4/80-FITC (1:100, clone BM8, Abcam #ab60343)
Rat mAb against mouse Cd206-Alexa Fluor® 647 (1:100, clone C068C2, Biolegend #141712)
Armenian Hamster mAb against mouse Cd11c-PE (1:100, clone N418, Biolegend #117308)
Rat mAb against mouse Ter119-FITC (1:100, clone Ter119, Biolegend #116205)
Rat mAb against mouse Cd19-FITC (1:100, clone 1D3/CD19, Biolegend #152403)
Armenian Hamster mAb against mouse TCRγ/δ-APC (1:50, clone eBioGL3 (GL-3, GL3), eBioscience # 17-5711-81)
Armenian Hamster mAb against mouse Cd3e-PE (1:50, clone145-2C11, eBioscience #12-0031-81)
Rat mAb against mouse Ly6G-BV711 (1:100, clone 1A8, Biolegend #127643)
Armenian Hamster mAb against mouse Cd11c-PE/Cyanine7 (1:100, clone N418, Biolegend #117318)
Mouse mAb against mouse Cd64-APC (1:50, clone X54-5/7.1, Biolegend #139305)
Rat mAb against mouse Cd45-FITC (1:100, clone 30-F11, Biolegend #103107)
Rat mAb against mouse Sca1-Pacific blue (1:100, clone D7, Biolegend #108120)
Rat mAb against mouse CD81-APC (1:100, clone Eat-2, Biolegend #104909)
Rat mAb against mouse Cd140α (Pdgfrα)-BB700 (1:100, clone APA5, BD Biosciences #742176)
Rat mAb against mouse Cd137-PE (1:100, clone 1AH2, BD Biosciences #558976)
Rabbit anti-mouse Tmem26 (1:200, Novus Biologicals #NBP2-27334SS)
PE-Cy7 goat anti-rabbit antibody (1:100, Santa Cruz Biotechnology #sc-3845)
Rat mAb anti-Cd45-Pacific blue (1:100, clone 30-F11 Biolegend #103126)
Rat mAb anti-Cd31-PE-Cy7 (1:50, clone 390, Biolegend #102418)
Syrian hamster mAb anti-Podoplanin (gp38)-APC (1:100, clone 8.1.1 Biolegend #127410)
Rabbit mAb anti-Cre recombinase (1:400, Cell Signaling Technology, D3U7F)
Goat anti-rabbit Alexa Fluor® 568 antibody (1:800, Thermo Fisher Scientific)
Rat mAb against mouse IL-5 APC (1:100, clone TRFK5, BD Biosciences #554396)
Rat mAb against mouse IL-13 PE-eFluor® 610 (1:100, clone eBio13A, eBioscience #61-7133-82)
Rabbit pAb against mouse MetEnk (1:300, Bioss Inc. #bs-1759R)
BV421 donkey anti-rabbit antibody (1:100, clone Poly4064, Biolegend #406410)

Antibodies for immunofluorescence, immunohistochemical staining and Western blot:
Rabbit pAb against mouse tyrosine hydroxylase (TH) (1:200, Merck Millipore #ab152); (1:2500 for Western blot analysis)
Rat mAb against mouse Cd3 (1:200, clone CD3-12, Abcam #ab11089)
Goat anti-rabbit Alexa Fluor® 488 (1:400, Thermo Fisher Scientific #A11008)
Goat anti-rat Alexa Fluor® 568 antibodies (1:400, Thermo Fisher Scientific #A11077)
Goat anti-rabbit Alexa Fluor® 568 antibody (1:800, Thermo Fisher Scientific #A11011)
Rabbit pAb against mouse UCP1 (1:500, Abcam, ab10983); (1:2500 for Western blot analysis)
Rabbit pAb anti-Cre (1:200, Novagen #69050)
Mouse mAb anti-FLAG (1:200, Sigma-Aldrich #F3165)
Rabbit mAb anti-perilipin (1:200, Cell Signaling Technology #9349S)
Rabbit pAb anti-β1-AR (1:200, clone V-19, Santa Cruz Biotechnology #C1313); (1:500 for Western blot analysis)
Rabbit mAb anti-β2-AR (1:200, clone EPR707(N), Abcam #ab182136); (1:2500 for Western blot analysis)
Goat pAb anti-IL-33 (1:200, R&D Systems #AF3626); (1:2500 for Western blot analysis)
F-actin fluorescent probe SPY555-actin (1:1000, Spirochrome #CY-SC202)
Syrian hamster mAb anti-gp38 (1:200, eBio8.1.1 (8.1.1), eBioscience #14-5381-82)
Rabbit pAb anti-Lyve1 (1:100, Abcam, ab14917)
Mouse mAb anti-β3-AR (1:1000, Santa Cruz Biotechnology #sc-515763)

	Rabbit pAb anti-HSP90 (1:2500, Cell Signaling Technology #4874S)<br>Rabbit mAb anti-GAPDH (1:2500, Cell Signaling Technology #2118L)
Validation	All antibodies are available on the manufacturer's websites. The antibodies have been validated by the manufacturers. No additional validation was carried out.

Animals and other research organisms

Policy information about studies involving animals; ARRIVE guidelines recommended for reporting animal research, and Sex and Gender in Research

Laboratory animals	Male and female C57BL/6N mice (obtained from the Laboratory Animal Unit at the University of Hong Kong) and male IL-33fl/fl-eGFP mice (imported from The Jackson Laboratory, Strain #030619) were used. All the mice were 8-week-old prior to starting the experiments. The age and sex of mice were indicated in each figure legend.
Wild animals	The study did not involve wild animals.
Reporting on sex	Adult male mice were used in all experiments except for the injection of LTβR-IgG2α to pharmacologically deplete iLN in the offsprings. Notably, the key findings of this study were also observed in female mice.
Field-collected samples	The study did not involve samples collected from the field.
Ethics oversight	All animal experiments were approved by the Committee on the Use of Live Animals in Teaching and Research (CULATR, #4375-17, #5184-19) at the University of Hong Kong.

Note that full information on the approval of the study protocol must also be provided in the manuscript.

Flow Cytometry

Plots

Confirm that:

- [x] The axis labels state the marker and fluorochrome used (e.g. CD4-FITC).
- [x] The axis scales are clearly visible. Include numbers along axes only for bottom left plot of group (a 'group' is an analysis of identical markers).
- [x] All plots are contour plots with outliers or pseudocolor plots.
- [x] A numerical value for number of cells or percentage (with statistics) is provided.

Methodology

Sample preparation	Fat tissues were minced into pieces with scissors before digesting in 2 mg/ml of Collagenase I buffer (DMEM supplemented with 3% of BSA) for 30 min at 37°C. Digested fat tissues were filtered through a 70 µm cell strainer and centrifuged at 800 g for 10 min at 4°C to separate stromal vascular fraction (SVF) from adipocyte fraction. The SVF pellets were incubated with red blood cell ammonium-chloride-potassium (ACK) lysis buffer for 1 min on ice, followed by centrifugation at 800 g for 10 min at 4°C and washed with PBS, before staining with respective flow antibodies.<br><br>Lymph nodes were dissected and disrupted using two 25 G needles before enzymatic digestion with DMEM medium containing 3.5 mg/ml Collagenase D and 40 µg/ml DNase I at 37°C for 30 min with agitation. The mixture was then filtered through a 70 µm cell strainer and centrifuged at 300 g for 5 min at 4°C. The cell pellet was resuspended and cultured in DMEM medium (supplemented with 10% FBS and 1% Penicillin/Streptomycin) (5% CO2, 37°C). After 24 hours, non-adherent cells were removed, and fresh medium was added to continue culturing until cells reached confluence. Adherent-stromal cells were then trypsinized and stained with respective antibodies.
Instrument	BD LSRFortessa Cell Analyzer, MoFlo Optical Bench Sorter (Beckman Coulter)
Software	FlowJo software version X.0.7 (Tree Star, Inc.)
Cell population abundance	More than 95% of the relevant cell populations within post-sort fraction. Purity mode was chosen during the process of cell sorting.
Gating strategy	The preliminary FSC/SSC gates of the starting cell populations were gated based on each cell population granularity and size. Dead cells and debris which have low FSC/SSC were excluded. Then using FSC-Height vs Area to enrich for single cells. Live cells were selected by cell viability dye. Gating strategies were drawn according to respective fluorescence minus one (FMO) controls to determine "positive" and "negative" staining cell populations.

- [x] Tick this box to confirm that a figure exemplifying the gating strategy is provided in the Supplementary Information.
